# Tcf12 is required to sustain myogenic genes synergism with MyoD by remodelling the chromatin landscape

**DOI:** 10.1038/s42003-022-04176-0

**Published:** 2022-11-09

**Authors:** Sheng Wang, Yinlong Liao, Haoyuan Zhang, Yunqi Jiang, Zhelun Peng, Ruimin Ren, Xinyun Li, Heng Wang

**Affiliations:** 1 Key Laboratory of Agricultural Animal Genetics, Breeding, and Reproduction of the Ministry of Education, College of Animal Science and Technology, Huazhong Agricultural University, Wuhan, China; 2 College of Animal Science and Technology, Shandong Agricultural University, Taian, China; 3 Department of Bioinformatics, Center for Translational Medicine, Naval Medical University, Shanghai, China

**Keywords:** Muscle stem cells, Muscle stem cells

## Abstract

Muscle stem cells (MuSCs) are essential for skeletal muscle development and regeneration, ensuring muscle integrity and normal function. The myogenic proliferation and differentiation of MuSCs are orchestrated by a cascade of transcription factors. In this study, we elucidate the specific role of transcription factor 12 (Tcf12) in muscle development and regeneration based on loss-of-function studies. Muscle-specific deletion of Tcf12 cause muscle weight loss owing to the reduction of myofiber size during development. Inducible deletion of Tcf12 specifically in adult MuSCs delayed muscle regeneration. The examination of MuSCs reveal that Tcf12 deletion resulted in cell-autonomous defects during myogenesis and Tcf12 is necessary for proper myogenic gene expression. Mechanistically, TCF12 and MYOD work together to stabilise chromatin conformation and sustain muscle cell fate commitment-related gene and chromatin architectural factor expressions. Altogether, our findings identify Tcf12 as a crucial regulator of MuSCs chromatin remodelling that regulates muscle cell determination and participates in skeletal muscle development and regeneration.

## Abstract (web summary)

TCF12 contributes to muscle development and regeneration and works with MYOD to govern myogenic genes at gene regulatory regions through chromatin remodelling

## Introduction

Muscle development and regeneration are sophisticated processes. During myogenesis, the muscle stem cells (MuSCs) are involved in various coordinated mechanisms including the proliferation and fusion of MuSCs to form multinucleated myotubes^1^. The molecular regulatory mechanisms of myogenic proliferation and differentiation rely on the activity of specific transcription factors (TFs)^2^. Several skeletal muscle-specific TFs, including MYOD, MYF5, MYOG and MRF4, direct the cell fate determination, proliferation and differentiation of myogenic precursors in multi-step processes that eventually lead to the formation of muscle fibers^3–5^. These factors function as heterodimers with E-protein subunits that bind to E-box consensus sites within the promoters and enhancers of genes, which are responsible for myogenic proliferation and differentiation during muscle formation and development^6–8^.

In addition to TFs, chromatin remodelling and transformation are also important in the proliferation and differentiation of MuSCs. During muscle development, the MuSCs would expand and fuse into myofibers in embryonic and postnatal muscle development^9–11^. In adults, MuSCs are located between the myofibers and basal lamina and could respond to the injury to regenerate the myofibers to restore muscle function. The activation and differentiation of MuSCs are frequently accompanied by the dynamic changes in higher-order chromatin structures, implying that the chromatin remodelling plays an integral role in myogenesis^12,13^. Given the critical role of chromatin restructuring in muscle cell specification, proliferation and differentiation, several chromatin architectural factors such as YY1, CTCF could precisely modulate the muscle specific TFs to reshaping the chromatin landscape^14–16^. In other words, the chromatin remodelling influences cell fate commitment and differentiation by activating myogenic regulatory factors (MRFs) to achieve skeletal myogenesis^2,17^. However, the effects of chromatin conformational changes on the fate determination of MuSCs and the underlying molecular mechanisms remain unclear.

During the activation and differentiation of MuSCs, chromatin conformational transitions affect cell fate decisions by regulating the expression of myogenic TFs and the accessibility of their binding sites. Tcf12 is known as the basic helix-loop-helix (bHLH) E-protein family member. And the binding of TCF12 to the E-box sites was affected by epigenome profiles, through which Tcf12 was involved in C2C12 myoblast differentiation; however, the underlying molecular mechanism remains unknown^18–20^. As a transcriptional regulator, Tcf12 participates in cell fate determination by regulating various lineage-specific gene expressions. Previous studies have demonstrated that Tcf12 acts as a cell-specification factor that integrates the epigenetic and transcriptional profiles of different cells during neurogenesis, haematopoiesis, adipogenesis and myogenesis^21–23^. Specifically, Tcf12 could dimerise with different protein partners and activate target gene transcription by binding to the E-box^22,24^. For instance, Tcf12 regulates the cell differentiation by interacting with TCF1 or acting as the TCF12/PRC2 complex in CD4^+^ CD8^+^ thymocytes and embryonic stem cells (ESCs) by forming heterodimers with other bHLH E-proteins^24,25^. In addition, it also plays specific roles in regulating cell fate determination, which promotes cell proliferation in hepatocellular carcinoma (HCC) and human ESCs (hESCs)^23^. Moreover, Tcf12 can bind to the E-box regions of MyoD and MyoG contributing to myogenesis; MyoD and MyoG are crucial factors responsible for myoblast proliferation and differentiation to form the skeletal muscle tissue^20,26–28^. Considering the interaction between TCF12 and MYOD, Tcf12 is predicted to impact the myogenesis of cultured myoblasts through chromatin remodelling. However, the underlying mechanisms of Tcf12 during muscle development and regeneration in vivo remain unclear.

In this study, we investigated the role of Tcf12 in skeletal muscle development and regeneration using two Cre drivers to induce conditional deletion of TCF12 in MuSCs: the Pax7Cre driver was used for early deletion of Tcf12 in MuSCs throughout the skeletal muscle lineage development, and the Pax7CreER driver was used for adult deletion of Tcf12 in MuSCs before and during muscle regeneration. RNA-seq, ChIP-seq (Chromatin Immunoprecipitation with sequencing) and ATAC-seq (assay for transposase-accessible chromatin with sequencing) were performed. In addition, the integrative analysis was performed to assess the correlation between chromatin accessibility and gene expression in TCF12-deficient and control cells. Further examination revealed that TCF12 deletion resulted in cell-autonomous defects during MuSCs proliferation and differentiation, indicating that TCF12 is a vital regulator of muscle development and regeneration. To reveal the molecular mechanisms underlying the abovementioned phenotypes, a comprehensive analysis of the TCF12 transcriptome and epigenome and its chromatin binding profile suggested that TCF12 interacted with MYOD to maintain chromatin stability and activate myogenic gene expression. TCF12 knockout resulted in aberrant chromatin opening and dysregulation of muscle cell fate commitment-related genes, which contributed to muscle development and regeneration defects.

## Results

### Loss of TCF12 in MuSCs causes defects in muscle development

**Fig. 1:**
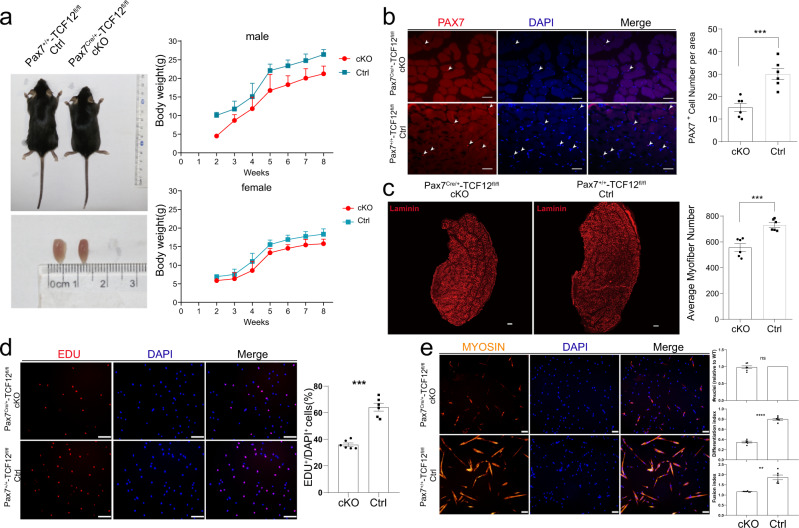
TCF12 deletion in MuSCs and muscle lineage cause defects in muscle development. (**a**) Representative images of the 2-month-old Pax7^+/+^-TCF12^fl/fl^ (Control, Ctrl) or Pax7^Cre/+^-TCF12^fl/fl^ (TCF12cKO, cKO) mice and images of their TA (tibialis anterior) muscles (left panel). Body weight of the Ctrl and TCF12cKO mice as recorded from 2 weeks after birth to 8 weeks (right panel); (**b**) Immunofluorescence (IF) staining for Pax7 and DAPI in the TA muscles of the TCF12cKO and Ctrl mice. Arrowheads point to the Pax7^+^ cells. Quantification of the Pax7^+^ MuSCs per area is shown on the right (*n* = 6); (**c**) IF staining for laminin in the TA muscles of the TCF12cKO and Ctrl mice; Quantification of average myofiber number is shown on the right (*n* = 6); (**d**) An equal number of isolated MuSCs from the TCF12cKO and Ctrl mice were cultured for 24 h and incubated with EdU for 2 h for immunostaining analysis (red). Quantification of the percentage of EdU-positive cells is shown on the right (*n* = 6); (**e**) Isolated MuSCs were cultured in a proliferation medium followed by 2-day differentiation. The extent of differentiation was visualized by IF staining of MYOSIN, and quantified by differentiation and fusion indexes. Total number of nuclei in both TCF12cKO and Ctrl were equivalent (*n* = 6). Scale bars = 100 μm. Data are expressed as the mean ± SEM. n.s. not significant, *P < 0.05, **P < 0.01, ***P < 0.001.

Constitutive ablation of TCF12 in mice results in a high percentage of postnatal death^29^, thus preventing detailed muscle analysis. To investigate the role of Tcf12 in skeletal muscle development, we used the Pax7Cre allele^30^ to delete Tcf12 in the myogenic lineage cells, including both MuSCs and myofibers. Mice with the conditional knockout allele Pax7^Cre/+^ and TCF12^f/f^ (hereafter referred to as TCF12cKO) as well as Ctrl mice with Pax7^+/+^ and TCF12^f/f^ were produced and used. The TCF12cKO mice were smaller than the Ctrl mice and exhibited no overt morphological deformity. The body weight of mice was recorded from 2 weeks after birth to 8 weeks (adult mice), and the size of TA muscle was found to be significantly decreased (Fig. 1a). To further assess the function of Tcf12 in muscle formation after TCF12 deletion in muscle tissue, we first confirmed the disruption of the Tcf12 locus based on genomic DNA detection (Supplementary Fig. 1a) and verified the diminishing TCF12 protein expression using western blot and immunostaining of the TA muscle (Supplementary Fig. 1b, c). In the TA muscle of 2-month-old TCF12cKO mice, the number of PAX7^+^ MuSCs significantly decreased as compared with the Ctrl mice (Fig. 1b). TCF12 deletion resulted in decreased myofiber number with similar myofiber size as detected by laminin immunostaining, which was consistent with the reduction in the TA muscle size (Fig. 1c; Supplementary Fig. 1d). Furthermore, MuSCs were isolated from the TCF12cKO mice for examining the specific role of TCF12 in myoblast proliferation and differentiation in vitro. The TCF12cKO derived MuSCs exhibited a significant decrease in the proliferation index as measured by EdU incorporation (Fig. 1d). In addition, myosin staining of myotubes in vitro revealed an impaired differentiation capability (Fig. 1e). Consistently, the western blot results revealed a reduction of the differentiation marker MYOG of limb muscles in the TCF12cKO compared with Ctrl mice (Supplementary Fig. 1e).

These results suggested that the specifically deletion of Tcf12 in Pax7 lineage muscle progenitors resulted in efficient TCF12 removal in muscle tissues and caused defects in muscle formation, highlighting the critical need for TCF12 in skeletal muscle development.

### TCF12 is required for skeletal muscle regeneration

**Fig. 2:**
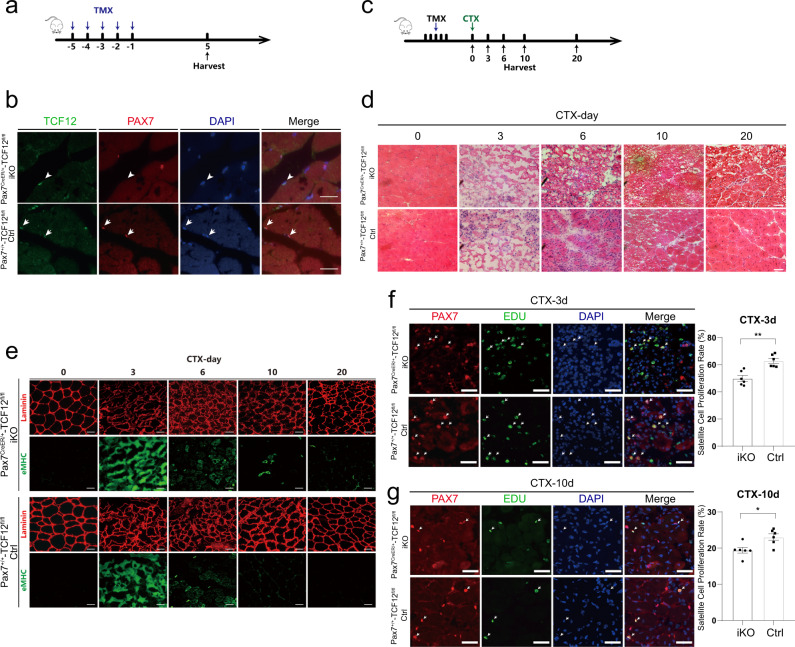
TCF12 is required for skeletal muscle regeneration. (**a**) Schematic outline of tamoxifen (TMX) administration in the Pax7^CreER/+^-TCF12^fl/fl^ (TCF12iKO, iKO) or Pax7^+/+^-TCF12^fl/fl^ (Control, Ctrl) mice; (**b**) IF staining for Pax7 and TCF12 on the TA muscles of the TCF12iKO and Ctrl mice; arrowheads point to the Pax7^+^/TCF12^+^ cells and arrows point to the Pax7^−^/TCF12^+^ cells; (**c**) Schematic outline of experimental design for evaluating the effect of TCF12 deletion on cardiotoxin (CTX)-induced muscle regeneration in the TCF12iKO mice; (**d**) H&E staining of the injured TA muscles collected from the designated time points after CTX injection to visualise the degree of regeneration; (e) IF staining for eMHC and laminin in TA muscle after CTX injury in the TCF12iKO and Ctrl mice; (**f**, **g**) IF staining for Pax7 and EdU on TA muscle 3 and 10 days after CTX injury. Arrows point to the Pax7^+^/EdU^+^ cells. Quantification of EDU-positive MuSCs is shown as the proliferation index on the right (*n* = 6); scale bars = 100 μm. Data are expressed as the mean ± SEM. n.s. not significant, *P < 0.05, **P < 0.01, ***P < 0.001.

To overcome the developmental defects, we used an inducible Cre driver in which tamoxifen (TMX)-inducible CreER protein is expressed from a modified Pax7 locus, Pax7CreERT2^31^. Mice with the Pax7^CreERT2/+^ allele were crossed with TCF12^f/f^ mice to generate Pax7^CreERT2/+^ TCF12^f/f^ mice (termed TCF12iKO) to permanently disrupt TCF12 function only in Pax7^+^ satellite cells upon TMX administration. The Pax7^+/+^ TCF12^f/f^ littermates treated with TMX were used as the Ctrl. After the administration of five consecutive doses of TMX, MuSCs were isolated and cultured to evaluate the deletion efficiency (Fig. 2a). Genomic DNA analysis revealed the disruption of TCF12 locus in the genome of the cultured MuSCs of the TCF12iKO mice (Supplementary Fig. 2a). Consistently, western blotting revealed successful depletion of TCF12 protein in the cells (Supplementary Fig. 2b). Immunofluorescence co-staining of PAX7 and TCF12 in TA muscle cross-sections indicated complete ablation of TCF12 protein in MuSCs at day 5 after TMX induction (Fig. 2b). Next, we used a cardiotoxin (CTX)-induced muscle injury regeneration model to assess the role of Tcf12 in muscle regeneration. The TA muscles of the TCF12iKO or Ctrl mice were subjected to a single CTX-induced injury and allowed to recover for 3-20 days before analysing the regenerated tissue (Fig. 2c). Both TCF12iKO and Ctrl mice exhibited normal muscle size and morphology before injury. However, CTX injection quickly induced extensive muscle damage and infiltration of inflammatory cells in the TA muscles of both TCF12iKO and Ctrl mice. TA muscles were harvested at different stages after injury. Muscle regeneration was severely disrupted in the TCF12iKO mice. Haematoxylin–eosin (H&E), laminin and eMHC staining indicated that MuSC descendants fused to form new myofibers in both the TCF12iKO and Ctrl mice. However, the regeneration of TA muscle of the TCF12iKO mice was severely delayed. At d10 after injury, the TA muscle was composed of degenerating myofibers, fibrotic tissues and inflammatory cells (Fig. 2d). The eMHC^+^ regenerating fibers were still easily identified until d10 in the TCF12iKO mice, whereas the Ctrl mice regenerated myofibers of larger size with diminished eMHC (Fig. 2e). Subsequently, EdU was intraperitoneally injected in mice a day before sample harvesting, and the number of EdU-positive MuSCs was counted to examine the proliferation capacity of TCF12iKO MuSCs. On day 3 after injury, more than 60% MuSCs were activated in the Ctrl mice, whereas less than 50% MuSCs were activated in the TCF12iKO mice (Fig. 2f). Similarly, EdU-positive MuSCs were significantly reduced in the TCF12iKO mice at 10 days after injury (Fig. 2g). Moreover, satellite cells were isolated from TA muscle 5 days after TMX induction to perform the proliferation and differentiation assays in vitro. TCF12 deletion obstructed MuSC proliferation, whereas the proportion of EdU-positive MuSCs decreased from 66% (WT mice) to 41% (TCF12iKO mice) after TCF12 knockout (Supplementary Fig. 2c). In addition, myosin immunostaining of 2-day differentiated MuSCs revealed that the cell differentiation capability was halted after Tcf12 knockout (Supplementary Fig. 2d). Altogether, Tcf12 deletion affected both MuSCs proliferation and differentiation, resulting in impaired muscle regeneration after injury.

### Activation of non-muscle gene programming in MuSCs with Tcf12 deletion

**Fig. 3:**
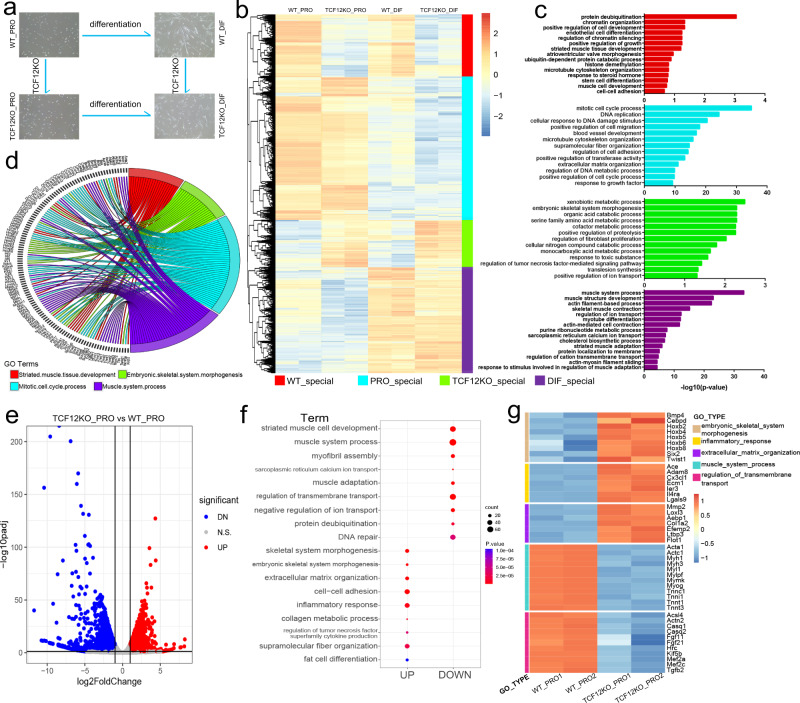
TCF12 deletion activates non-muscle gene programming in MuSCs. (**a**) Schematic outline for comparing the gene expression patterns between different types of muscle cells, including Ctrl (WT_PRO and WT_DIF) and TCF12KO (TCFKO_PRO and TCFKO_DIF) cells; (**b**) Heatmap demonstrating different transcriptional patterns in each cell type after cluster analysis. Only genes expressed at least in one stage or group (TPM ≥ 1) were included; Note the WT special (red), TCF12KO special (green), proliferation special (blue), differentiation special (purple) upregulated genes were marked; (**c**) GO analysis of upregulated genes in the four types of cell-to-cell comparisons; (**d**) Circus plot showing the cell-specific biological process-related genes; the selected DEGs revealed the aberrant induced biological processes in muscle cells after Tcf12 deletion, especially the functions involved in embryonic skeletal system morphogenesis; (**e**) Volcano plot of the RNA-seq data of TCF12KO_PRO versus WT_PRO cells. Significantly upregulated (red) and downregulated (blue) genes are highlighted (|logFC|> 2, *P* value < 0.01); (**f**) GO analysis of significantly dysregulated genes in WT_PRO and TCFKO_PRO cells. Biological processes were selected based on the count of gene number (gene count > 10) and *P* value (*P* value <1 × 10^−10^); (**g**) Heatmap with the normalized reads per gene of WT_PRO and TCFKO_PRO cells.

To assess the underlying mechanisms of impaired myogenesis in MuSCs with Tcf12 deletion, transcriptomes were analysed in both proliferating (PRO) and differentiating (DIF) cells isolated from the Ctrl and TCF12KO mice (Fig. 3a). PCA was performed and the correlation between replicates was higher than 97% within each sample (Supplementary Fig. 3c, d). The transcriptome profiles of four cell types, namely, WT_PRO, TCFKO_PRO, WT_DIF and TCFKO_DIF, were clustered to form distinguished expression patterns, which were exhibited using a heatmap (Fig. 3b; Supplementary Data 2). Furthermore, we identified differentially expressed genes (DEGs) (>2-fold) between different samples and identified the cell-specific DEGs that exhibited the highest expression in WT_PRO, TCFKO_PRO, WT_DIF or TCFKO_DIF cells, respectively (Supplementary Data 3). Gene Ontology (GO) functional analysis of the cell-specific DEGs was used to characterise different cell types (Fig. 3c). At the proliferation stage, the biological processes such as “DNA replication”, “mitotic cell cycle”, “microtubule cytoskeleton” and “extracellular matrix organisation”, which are involved in cell proliferation and development, were prevalent in both WT_PRO and TCFKO_PRO cells (PRO_special). Comparably, the myofiber formation- and biosynthetic metabolism-related processes were found in the upregulated genes in both WT_DIF and TCFKO_DIF myotubes (DIF_special), confirming the distinct cellular states between proliferating and differentiated cells. However, compared with the knockout cells, WT_special (WT_PRO, WT_DIF) cells exhibited a high expression of genes that were enriched in “striated muscle tissue development”, “chromatin organisation” and protein de-ubiquitination. These genes were subsequently downregulated upon TCF12 ablation. In contrast, genes involved in embryonic morphogenesis and lipid biosynthesis processes were significantly upregulated after Tcf12 deletion in both myoblasts and myotubes (TCFKO_PRO and TCFKO_DIF, TCFKO_special) (Fig. 3d).

Subsequently, DEGs were analysed in the four cell types. Tcf12 deletion resulted in major transcriptomic changes in proliferating cells; a total of 1977 DEGs were identified between WT_PRO and TCFKO_PRO cells as opposed to 655 DEGs detected between WT_DIF and TCF12KO_DIF cells (Fig. 3e; Supplementary Fig. 3e). These results suggested that Tcf12 deletion caused more changes in gene expression and biological processes in myoblasts than in myotubes, indicating that Tcf12 played a major role in the proliferation of myogenic cells. Therefore, transcriptional profiles between WT_PRO and TCFKO_PRO were subsequently scanned. GO analysis revealed that 1416 downregulated genes in the TCF12KO myoblasts were enriched in striated muscle cell development, myofibril assembly and protein de-ubiquitination, indicating that Tcf12 is essential for maintaining myogenic gene expression. In contrast, 561 upregulated genes were enriched in many non-muscle processes including embryonic skeletal system morphogenesis and collagen metabolism (Fig. 3f). Inflammatory response-related genes, including positive regulators of apoptosis, were also upregulated in TCF12KO myogenic cells (Fig. 3g). However, the apoptosis analysis by annexin V staining and sorting showed no apparent difference between WT and TCF12KO cells (Supplementary Fig. 3j). Furthermore, the expression levels of apoptosis and senescence marker genes were comparable between the two groups of cells^32,33^ (Supplementary Fig. 3h, 3i). Interestingly, the quiescent marker genes, such as Pax7, Sdc3, Spry1 and Cd34, Notch1 and Notch3^34–36^, were all upregulated in the cells upon TCF12 deletion, suggesting the Tcf12 deletion may drive the cells toward to a quiescent state (Supplementary Fig. 3k).

Altogether, these results suggested that Tcf12 was required to sustain myogenic gene expression and repress the developmental potential of non-muscle lineages, thus making it an essential TF for maintaining myogenic capability at the transcriptome level.

### Aberrant chromatin opening in TCF12KO cells

**Fig. 4:**
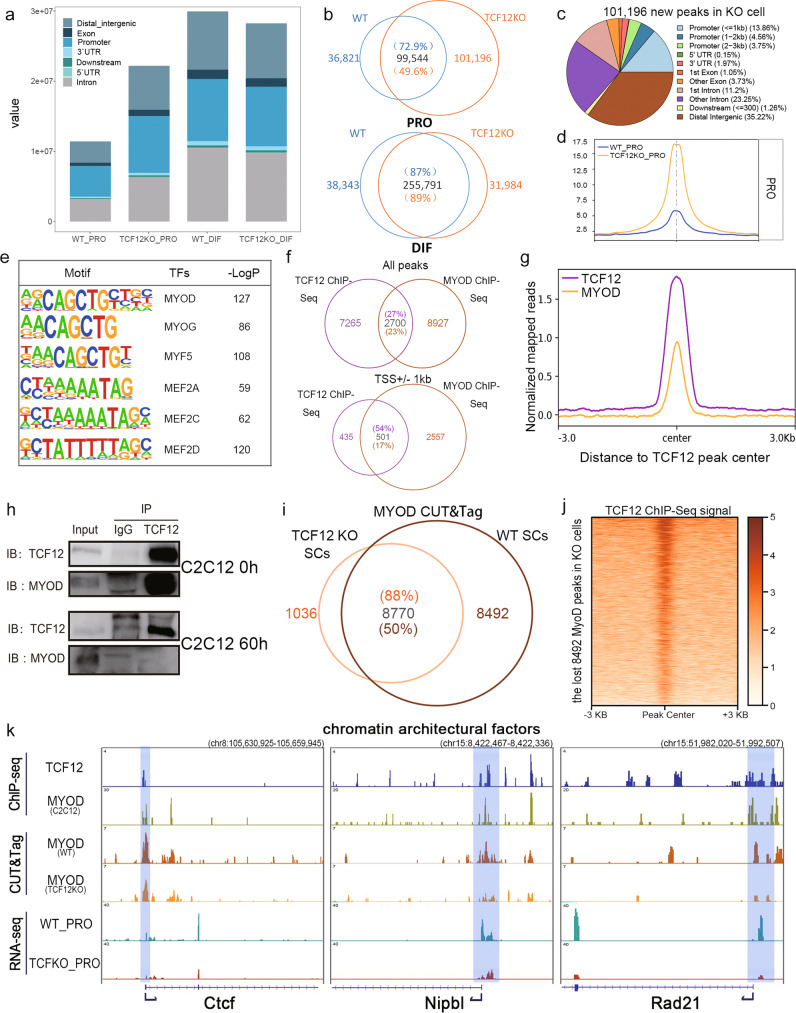
Aberrant chromatin opening occurred in TCF12KO cells. (**a**) Percentage of the total number of ATAC-seq peak signals distributed in distinct genomic regions as indicated in the four cell types; (**b**) Venn diagram of the ATAC-seq peaks in the four cell types. Overlap peaks of proliferation (top) and differentiation (bottom) cells in WT (blue) and TCF12cKO (orange) were illustrated, overlapped regions were assigned in grey; (**c**) Distribution of 101,196 regions identified upon TCF12 ablation, which are annotated using ChIPseeker; (**d**) ATAC-seq average signal profiles of isolated PRO cells in the Ctrl (blue) and TCF12cKO (orange) mice; (**e**) TF motif enrichment identified from ATAC-seq peaks of additional 101,196 open chromatin regions; (**f**) Venn diagram representing the overlap of TCF12 and MYOD ChIP-seq peaks across the genome (top) or around ±1 kb of the TSS region (bottom); (**g**) Average signal profiles of TCF12 (ratio to input; purple) and MYOD (ratio to input; orange) ChIP-seq intensities at TCF12 peaks; (**h**) Co-IP analysis showing TCF12 interacts with MYOD in C2C12 myoblasts and myotubes. Lysates from C2C12 myoblasts (0 h) and myotubes (60 h) subjected to Co-IP assays with anti-TCF12 or anti-IgG antibodies and blotted with anti-MYOD and anti-TCF12 antibodies; (**i**) Venn diagram of the MYOD CUT&Tag peaks in TCF12KO and WT myoblasts. Overlap peaks in TCF12KO (orange) and WT (brown) cells were illustrated, overlapped regions were assigned in grey; (**j**) Heatmap to illustrate the specific TCF12 ChIP-seq signal at the 8492 lost MYOD binding sites in KO cells; (**k**) IGV browser examples of chromatin architectural factors bound by TCF12 and MYOD that were downregulated upon TCF12 ablation. Genome tracks of TCF12 and MYOD ChIP-seq (top), CUT&Tag of MYOD in WT and TCF12KO myoblasts (medium) and RNA-seq of WT_PRO and TCFKO_PRO cells (bottom).

Because gene expression occurs within accessible chromatin regions, we performed ATAC-seq in primary myoblasts and myotubes in the Ctrl and TCF12cKO mice to assess dynamic changes of the chromatin accessibility upon Tcf12 deletion. First, the genomic distribution of the open chromatin regions was examined in TCF12-deficient and Ctrl cells. We found that TCF12 deletion induced much more dramatic changes in chromatin accessibility in PRO cells than in DIF cells (Fig. 4a). Approximately half of the open accessible chromatin regions (50.4%) in the TCF12KO PRO cells were newly formed after TCF12 deletion, whereas only 11.12% open chromatin regions were newly formed in TCF12KO DIF cells (Fig. 4b; Supplementary Fig. 4c). Subsequently, we focused on TCF12-deficient and Ctrl PRO cells and examined the 101,196 open chromatin regions identified as additional open regions upon Tcf12 ablation. TCF12 deletion-induced open chromatin regions were mostly located at distal intergenic (35.22%), intronic (34.45%) and promoter (22.17%) regions (Fig. 4c). In addition, the ATAC-seq signal at the TSS region across the genome of the TCF12KO and WT cells confirmed the elevated chromatin accessibility at gene promoter regions (Fig. 4d).

Since the TFs may regulate the chromatin organisation and activity during cell commitment and differentiation, we hypothesized that the specific TF-binding motifs associated within the altered open chromatin regions could provide us the clues for TF-induced chromatin remodelling. We used Homer to identify the known TF-binding sites within the newly formed 101,196 open chromatin regions in TCF12KO cell. The TFs whose binding sites enrichment were altered during TCF12-deletion induced chromatin opening were considered candidate regulators of chromatin remodelling. The binding motifs of several muscle regulatory factors (MRFs) were identified across the open chromatin regions, and their motif accessibility was increased upon Tcf12 deletion (Fig. 4e). It indicated the TCF12 may regulate the chromatin binding dynamics of these MRFs and the loss of TCF12 leads to irregular MRF binding to the DNA and the followed aberrant chromatin opening. Thus, we predict that TCF12, as a bHLH co-transcription factor, can cooperate with other MRFs such as MyoD to maintain the epigenetic landscape of muscle cells.

Therefore, we further investigated whether TCF12 could work in association with MYOD by analyzing the ChIP-seq data of the two factors. Significant co-occupancy of TCF12 and MYOD signals were detected across the genome in myoblasts. For instance, 54% of the TCF12 target genes were also bound by MYOD, suggesting that TCF12 and MYOD worked together to regulate the target gene expression (Fig. 4f, g). Co-IP results further confirmed that TCF12 indeed physically interacted with MYOD in myoblast and MYOG in myotubes, separately (Fig. 4h; Supplementary Fig. 5a). Furthermore, we also found that TCF12 could bind to the TSS region of MyoD and MyoG (Supplementary Fig. 5b, c). Interestingly, according to the Co-IP results of MYOD and TCF12 in myotubes, TCF12 does not interact with MYOD in differentiated cells. We further analyzed the ChIP-seq data of TCF12 and MYOD in myotubes and found only 578 overlapped peaks (less than 5%) between the two factors (Supplementary Fig. 5d, e). It indicated that TCF12 interact with the MYOD in proliferating but not differentiated cells.

According to our result, TCF12 and MYOD worked together specifically in proliferating cells; and aberrant chromatin opening was also observed upon TCF12 deletion in myoblasts. So we hypothesized that it is the TCF12/MYOD heterodimer abrogation in the TCF12 KO cells led to more chromatin openness. Therefore, we examined the alterations of MYOD binding sites upon TCF12/MYOD disruption by CUT&Tag in WT and TCF12KO myoblasts. As expected, the MYOD binding sites reduced by ~50% after TCF12 ablation (Fig. 4i). In retrospect, the lost MYOD binding sites in KO cells were overlapped with TCF12 peaks in WT cells (Fig. 4j), indicating that TCF12 is necessary for proper MyoD binding. Genes associated with these lost MYOD binding sites were mainly enriched in “establishment of cell polarity” and “muscle structure development”, which were necessary for muscle development (Supplementary Fig. 5f). In addition, the expression of several chromatin conformational factors was also detected to be downregulated upon TCF12 deletion. Accordingly, the MyoD binding was also reduced at the TSS region of these CTCF and cohesin formation-related genes (Fig. 4k). Because Ctcf, Nipbl and Rad21 are vital for the formation of the topologically associated domains (TADs), it suggested that TCF12 participated in chromatin organization regulation through the heterodimerization with MYOD. According to previous studies, knockdown of cohesin formation increased chromatin accessibility; disruption of CTCF would also repress polycomb-repressive-complex 2 (PRC2) binding in the orchestration of myogenesis^37–39^. It indicated that the aberrant chromatin opening and the downregulation of these chromatin regulators was caused by TCF12 ablation and the accompanied reduction of MYOD activity.

Altogether, the above results suggested that through interactions with MYOD, TCF12 was able to stabilize chromatin conformation and maintaining myogenic gene expression by regulating chromatin architectural factors, particularly in the proliferating MuSCs.

### TCF12 and MYOD guide muscle development through cell fate commitment

**Fig. 5:**
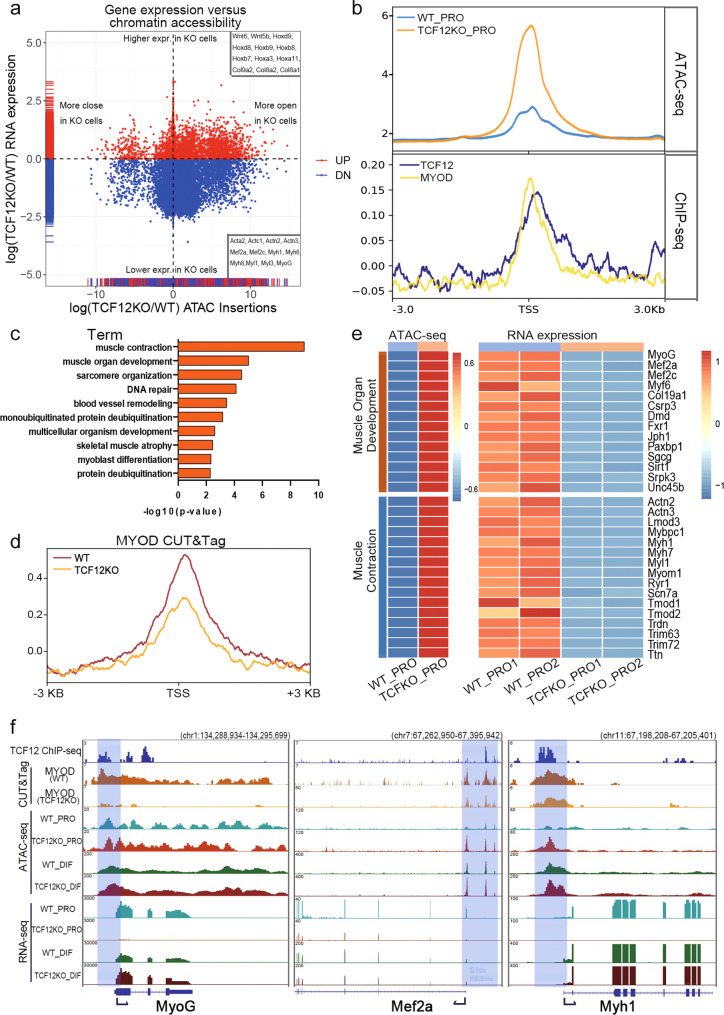
TCF12 and MYOD maintained muscle development through cell fate commitment. (**a**) Scatterplot showing the fold change of ATAC signals (TCF12KO/WT) versus RNA expression (TCF12KO/WT) in myoblasts. Each dot represents a gene identified in RNA-seq; (**b**) Average signal profiles of ATAC-seq (top) and ChIP-seq (ratio to input; bottom) data. The ATAC-seq profiles of those downregulated genes in WT_PRO (blue) and TCFKO_PRO (brown) cells, which accompanied with elevated peaks at TSS region upon TCF12 ablation. TCF12 (dark blue) and MYOD (yellow) ChIP-seq profiles showed significant TCF12 and MYOD binding peaks at the TSS region of the downregulated genes; (**c**) GO analysis for downregulated genes accompanied by elevated peaks at the TSS region. Biological processes were selected based on gene number (gene count >10) and *P* value (*P* value < 0.01); (**d**) MYOD CUT&Tag average signal profiles (ratio to input) of the TCF12/MYOD target genes in WT (brown) and TCF12KO (orange) cells; (**e**) Heatmap with the normalized reads per gene of both WT_PRO and TCFKO_PRO RNA-seq and ATAC-seq data of different gene clusters; (**f**) IGV browser examples of cell-fate commitment-related genes bound by TCF12 and MYOD, showing genome tracks of TCF12 ChIP-seq (top), CUT&Tag of MYOD in WT and TCF12KO myoblasts (middle), ATAC-seq of four cell types (middle) and RNA-seq of four cell types (bottom).

To understand the underlying mechanism of impaired myogenesis and the accompanied aberrant chromatin opening caused by TCF12 ablation, we further analysed the transcriptome profiles with the corresponding ATAC-seq data in TCF12-deficient and Ctrl myogenic cells. ATAC-seq average signals were calculated for both upregulated and downregulated genes of the TCF12KO versus Ctrl myoblasts (Fig. 5a). Among all expressed genes, the majority of the upregulated genes (76%, 1836/2404) were marked with elevated chromatin accessibility at the TSS region upon TCF12 ablation, indicating that these genes were activated through the opening of condensed gene regulatory regions. Many of these genes were identified as non-muscle genes involved in embryonic morphogenesis, such as the Hoxb family, resulting in impaired myoblast differentiation (Supplementary Fig. 6c). Intriguingly, only approximately 40% downregulated genes exhibited reduced chromatin accessibility around the TSS region after Tcf12 ablation; whereas the remaining 60% downregulated genes exhibited increased chromatin accessibility. We hypothesized other underlying mechanisms may contribute to the blocking of gene transcription even the chromatin structure was permissive for the expression of these genes.

The epigenetic landscape and transcriptome profiles were significantly changed resulted from TCF12 ablation; hence, we investigated the TCF12 and MYOD co-binding sites in proliferating cells according to the TCF12/MYOD interaction. CUT&Tag of MYOD in WT and TCF12KO myoblasts was also used to validate the molecular mechanisms of TCF12/MYOD interaction. We found that both TCF12 and MYOD binding signals were enriched at the TSS region of the downregulated genes, which were identified as the target genes of TCF12 and MYOD (Fig. 5b). These results indicated that Tcf12 deletion disrupted the cooperative activation of TCF12 and MYOD targeted genes, although the local chromatin structure remained permissive for gene transcription. In addition, the GO analysis of downregulated genes with higher chromatin accessibility after TCF12 deletion revealed that the genes were enriched in the pathways of muscle construction and muscle organ development (Fig. 5c). Moreover, using CUT&Tag of MYOD, significant MYOD decline were detected at the TSS of these TCF12/MYOD target genes (Fig. 5d). Thus, it indicated that it is the diminished binding of MYOD at the TSS of these TCF12/MYOD target genes that downregulated the gene expression after TCF12 deletion. We carefully examined representative genes from each GO term and confirmed the seemingly opposite regulation between chromatin accessibility and gene expression. Upon TCF12 ablation, MYOD reduction downregulated the expression of important myogenic TFs, such as Mef2a, MyoG, and myofiber formation genes, Myh1, Myh7, and Actn2 (Fig. 5e, f). These results demonstrated that myogenic transcription was severely compromised owing to Tcf12 deletion and the accompanied reduction of MYOD activity with respect to chromatin accessibility.

Altogether, our data demonstrated that TCF12 and MYOD worked together to maintain the chromatin landscape in myogenic cells to promote the expression of myogenic genes. Upregulated genes were found in the open chromatin regions; in addition, myogenic differentiation capability was suppressed owing to Tcf12 deletion and MYOD decline, resulted in apparent chromatin conformational disorder and further leading to defects in skeletal muscle development and regeneration.

## Discussion

Myogenesis requires a complex orchestration of multiple TFs and coordinates with precise transcriptional and epigenetic programming. As a class I basic-HLH superfamily protein, Tcf12 achieves ever-elaborating activities required for lineage specification by regulating the expression of a restricted set of tissue-specific target genes^19,22,40^. In this study, we elucidated Tcf12 function in muscle development and regeneration using diverse genetic mouse models. Phenotypic characterisation revealed that muscle-specific Tcf12 deletion significantly impaired muscle development. Body weight was profoundly decreased after Tcf12 knockout, and the TA muscle size was smaller in knockout mice than in WT mice. TCF12 deficiency caused an intrinsic delay in MuSCs proliferation and differentiation, thereby impairing the regenerative capacity of knockout mice and highlighting the critical role of TCF12 in regulating muscle regeneration. Moreover, the cell fate upon TCF12 deletion was examined and we found that the KO cells were not undergoing apoptosis or senescence. So we further performed RNA-seq and ATAC-seq to assess the potential molecular mechanisms of Tcf12 in regulating MuSCs proliferation and differentiation. Genome-wide ChIP-seq analyses revealed that TCF12 co-localized with MYOD to regulate chromatin accessibility and transcriptional profile by participating in cell fate determination during myogenesis. Altogether, the TCF12/MYOD complex may act as an epigenetic factor, thus stabilizing chromatin accessibility and promoting developmental myogenesis.

Previous studies have demonstrated that Tcf12 plays a crucial role in regulating cell growth and differentiation during embryonic development. However, the role of Tcf12 in myogenesis remains unclear^23^. Known as the bHLH E-protein, E12 (TCF3), E47 (TCF3) and HEB (TCF12) were both reported dimerizing with MRFs in C2C12 cells^41^. It was demonstrated that TCF3 was able to heterodimerize with MYOD in vivo using fluorescence resonance energy transfer (FRET), a technique for evaluation of protein-protein interactions^42,43^. TCF12 was also dimerized with MYOD in promoting cell proliferation and obstructing cell differentiation^20^. Furthermore, Tcf12 and Tcf3 proteins would bind to a similar spectrum of E-box sequences as homo-oligomers, by which MRF/TCF complexes were critical to activating terminally differentiation during muscle development^20,26,41^. In addition, Tcf12 affects cell fate decisions in different cell types; for instance, it can improve cell proliferation in HCC cells. In this study, the proliferation rate of MuSCs isolated from the TCF12iKO and TCF12cKO mice was dramatically decreased and the differentiation capability was suppressed. Tcf12 deletion not only caused a reduction in the body weight of mice but also induced a decline in the number of myofibers, demonstrating the crucial role of Tcf12 in myogenic cell development. Moreover, we identified that TCF12iKO mice displayed severe regeneration retardation after acute injury, which is attributed to an intrinsic delay in MuSC pool expansion. Therefore, Tcf12 affected muscle phenotype in terms of the number of myofibers during muscle development and regeneration.

To assess the mechanisms underlying the delayed proliferation of TCF12KO cells, we performed transcriptome analysis to identify the gene expression patterns between TCF12KO and control myogenic cells. TCF12 deficiency elevated developmental regulatory gene expression and repressed myogenic gene expression. Several previous studies have revealed the effects of TCF12 on determining cell fate in different cell types. In hESCs, the double deletion of TCF3 and TCF12 resulted in worse deficiencies during neural ectoderm fate determination^23^. TCF12 acts as a bHLH protein and dimerizes with TWIST1 to maintain the progenitor state of stem cells and block the entry into the endodermal lineage^21^. TCF12 also regulates developmental fates associated with PRC2 and SMAD2/3, encompassing mesodermal and endodermal development^24^. Our study revealed that TCFKO_special genes were enriched in the cell specification process, especially in proliferation status, which upregulated after TCF12 ablation, including Hox, Fox and Wnt gene families^24^. However, WT_special genes, which were subsequently downregulated in TCF12-deficient cells, were predominantly enriched in muscle system processes, including the development of rhabdomyocytes and regulation of transmembrane transport. In addition, our results even indicated the potential functional compensation between E-proteins in myogenic differentiation, since the class I E-protein Tcf3 was slightly upregulated after Tcf12 knockout (Supplementary Fig. 3f). As highly evolution conserved, Tcf12 and Tcf3 were structurally and functionally related^44^. Since Tcf12 and Tcf3 proteins were reported to bind to the similar spectrum of E-box sequences as homo-oligomers, genes regulated by Tcf3 would also demonstrate depositing with TCF12 binding signals^20,26,41^. So Tcf3 could potentially compensate for the ability of Tcf12 to regulate cell proliferation and differentiation. Furthermore, a series of translational and post-translational modifications were also reported to regulate myogenesis^45^. Unlike the equal MyoG mRNA expression in WT and TCF12KO myotubes, the protein was impaired upon TCF12 deletion in limb muscle. We found that Eif4ebp1, a translation repressor protein, was specifically upregulated after TCF12 ablation in myoblasts and myotubes^46^ (Supplementary Fig. 3g). We hypothesized that translational and post-translational mechanisms may occur during MYOG protein synthesis, which could contribute to the downregulation of MYOG protein in TCF12KO muscle tissue. These findings demonstrated that TCF12 was responsible for the expression of myogenic genes, and its absence caused defects in muscle development. Because TCF12 is an integral component of various stem cell types, it is considered a pivotal TF in the regulation of muscle development and regeneration.

Epigenetic regulation is another layer of molecular mechanism that regulates gene expression by altering chromatin accessibility in different cell types. In CD4^+^ CD8^+^ thymocytes, TCF12 functionally cooperates with TCF-1 for defining the epigenetic transcriptional status^25^. We found that Tcf12 deletion profoundly enhanced the chromatin accessibility of MuSCs, especially for the chromatin regions harboring multicellular organism development- and cell adhesion-related genes. Because gene expression occurs within accessible chromatin during muscle development and cell specification, epigenetic mechanisms modulate muscle gene expression and on the previous work that addresses three-dimensional genome architecture to achieve skeletal myogenesis by affecting cell-fate determination and differentiation^2^. Our results suggested that TCF12KO-induced chromatin accessibility alterations were associated with the downregulation of 60% genes that were deposited with the TCF12 and MYOD ChIP-seq peak signals. These downregulated genes caused by MyoD binding depletion even showed modest chromatin opening, including important myogenic TFs MyoG and Mef2a, were important regulators in muscle formation. Our results demonstrated that Tcf12 knockout impeded MuSC-fate decisions during early muscle development, which is consistent with the findings of previous studies implicating Tcf12 as a potent pro-differentiation factor during myogenesis^20^.

In addition to directly controlling the expression of cell specification genes, TCF12 also modulates muscle organ development by stabilising MYOD protein. Studies have reported that Tcf12 dimerizes with different protein partners to participate in tissue development^19,21,23^. Because MYOD localises on the anchors of chromatin loops in muscle cells, it can induce the circularisation of linear DNA to confirm MyoD-orchestrated chromatin looping^16^. Moreover, MyoD^-/-^ muscles were reported to have severe regeneration deficit and deteriorated MuSCs differentiation capacity^47^. Therefore, we hypothesized that TCF12 and MYOD acted as epigenetic controllers to stabilize chromatin conformation and facilitate the proliferative growth of MuSCs. Moreover, the locations bound by both TCF12 and MYOD were specifically around the promoter region, as detected using H3K27ac data in C2C12 myoblasts^48^ (Supplementary Fig. 4f). TCF12/MYOD shared binding sites for genes involved in cell lineage specification and chromatin architectural modelling, including Ezh2, Suz12, Uty/Utx, Nipbl and Ctcf, and the expression levels of these genes were decreased to reshape the chromatin landscape after Tcf12 deletion. It showed that all these downregulation of chromatin regulators, such as Ctcf, Nipbl, and Rad21, could be caused by the significant decreased MYOD binding at the TSS region upon TCF12 depletion.

Furthermore, we hypothesized that the downregulation of chromatin regulators could be potentially involved in the process of aberrant chromatin opening. Known as cohesin core complex subunits RAD21 and its modulators NIPBL, the downregulation of these genes was implicated in the disruption of cohesin formation^49^. The knockdown of cohesin could increase chromatin accessibility in immature human hematopoietic stem and progenitor cells^37^. In addition, recruiting of EZH2 and SUZ12, the components of PRC2, were required for CTCF dependent chromatin organization^39,50^. Thus, the downregulation of EZH2, SUZ12, and even CTCF in TCF12KO cells would also re-organize the chromatin that may associate with chromatin de-repression and aberrant opening^38^. It indicated that TCF12/MYOD would co-regulate the cohesin formation and even the stability of chromatin conformation to regulate the muscle cell fate determination.

In conclusion, TCF12 coordinates with MYOD to exert chromatin regulation; precise transcriptional and epigenetic remodelling was also observed after Tcf12 knockout. Chromatin accessibility of the TSS region of TCF12- and MYOD-related genes in myogenic cells was enhanced in the absence of TCF12, particularly for myogenic determination- and chromatin conformational remodelling-related genes. In addition, Tcf12 deficiency is responsible for the disruption of the TCF12/MYOD complex, thus triggering the downregulation of cohesin formation- and myogenesis-related genes due to the reduction of MYOD activity. Therefore, Tcf12 deletion remodels the chromatin landscape and transcriptional profiles to regulate the myogenic specification of MuSCs.

## Methods

### Mice

This study was conducted in accordance with the protocols approved by the Hubei Province Committee on Laboratory Animal Care: HZAUMO-2021-0188. Tcf12tm3Zhu (# 024511), Pax7tm1 (cre) (#010530) and Pax7tm1cre/ERT2 (#017763) mice were purchased from Jackson Laboratory. Male and female mice were used in Fig. 1a, and male mice were used in all the other experiment. The mice were genotyped by PCR of tail DNA following standard protocols. The primers used for genotyping and deletion detection are as follows: genotyping-F: CTCATTCTTCATCAGGCCGTG, genotyping-R: AAGCCACATTTATTGATTCCT; deletion-F: AGCACTTAGTACATTTGAATCAGT, deletion-R: CCAAAGCCACATTTATTGATTCCT. The mice were fed and maintained in an animal facility, with no specific pathogen. All procedures involving mice were approved by the HZAU University Animal Care and Use Committee.

### Isolation and culture of MuSCs

MuSCs were isolated from the hindlimb muscle tissues of 8-week-old mice as previously described^51,52^. The muscle tissues were digested with collagenase I (2 mg/ml; Sigma, USA, C1639) at 37 °C. The dissociated suspension was sifted through 100, 200, and 400 mesh sieves. Then, the suspension was washed by primary myoblast growth medium (RPMI-1640 medium (Life, USA) supplemented with 15% fetal bovine serum (FBS; Gibco, USA, 10082-147), 1% non-essential amino acids (NEAA; Gibco, USA), 0.5% chicken embryo extract (CEE; GEMINI, USA, 100-163p), 1% GlutaMax (Gibco, USA), 1% Antibiotic-Antimycotic (Gibco, USA) and 2.5 ng/mL of bFGF (Life, USA, 13256-029)). The suspension was cultured in uncoated plates for 2 h cell purification^52^. Fresh isolated cells were prepared for subsequent RNA-seq and ATAC-seq experiments. MuSCs isolated from wildtype (WT) and TCF12 knockout (TCF12KO) mice were cultured on Matrigel-coated (BD, USA, 356234) plates after purification at 37 °C in primary myoblast growth medium. Furthermore, the purity of isolated MuSCs were confirmed to be higher than 90% (*n* = 3) (Supplementary Fig. 3a). Then, the differentiation was induced using the differentiation medium composed with Dulbecco’s Modified Eagle’s Medium (DMEM; Gibco) and 2% horse serum (Gibco).

### Cell lines

C2C12 cells were used for TCF12 ChIP-seq and Co-IP validation for TCF12 and MYOD, MYOG. C2C12 cells were purchased from ATCC. C2C12 cells were cultured at 37 °C in DMEM medium supplemented with 10% FBS and 1% Antibiotic-Antimycotic and were induced to differentiation using DMEM medium supplemented with 2% horse serum.

### Apoptosis detection

Cells were counted and collected for apoptosis detection using Annexin V-FITC/PI Apoptosis Detection Kit (Yeasen, 40302ES20) according to the manufacturer’s instructions. Briefly, after digestion with EDTA-free trypsin, cells were centrifuged at 300 °C for 5 min. After washed with pre-chilled PBS twice, cells were resuspended with 100 μL Binding Buffer. Add 5 μL Annexin V FITC and 10 μL PI Staining Solution, cells are mixed gently and then reacted at room temperature for 15 min avoiding light. Add 400 μL of 1×Binding Buffer, cells are mixed and placed on ice. Flow cytometry (BD, BD FACS Aria II) was used for apoptosis detection. Data analysis was carried out with FlowJo software.

### EdU treatment

EdU was added to the growth medium 2 h before fixing the cells, and EdU-labelled cells were visualized using the Alexa Fluor 488-conjugated azide (Invitrogen, A20012).

### In vivo treatment

Tamoxifen (TMX; Sigma, T5648) was dissolved in corn oil and administered intraperitoneally at a dose of 2 mg per day per 20 g of body weight for 5 consecutive days to induce TCF12 deletion. A cardiotoxin analogue (CTX; MCE, HY-P1902A) was used to induce muscle regeneration; 50 μL of the 10 μM solution was injected into the tibialis anterior (TA) muscle of 8-week-old mice. A day before harvesting, the mice were administered 5 mg/kg of EdU (Invitrogen, E10187) intraperitoneally as described by the supplier. Subsequently, the TA muscle was dissected and fixed in 4% paraformaldehyde (PFA), followed by freezing of the sections and immunostaining.

### Immunoblotting and immunofluorescence staining

For western blotting, MuSCs were digested using 0.25% trypsin and lysed using RIPA buffer, and total protein concentration was measured using a BCA kit (Beyotime, P0010S). Proteins were separated by SDS-PAGE; subsequently, the separated proteins were blocked using 5% fat-free milk for 2 h at room temperature. After incubating the proteins with primary antibodies at 4 °C, secondary antibodies were added, and the protein complex was visualized. The following antibodies were used: HEB (A-6) (Santa, 1:500), β-tubulin (ABclonal, 1:1000), HRP-labelled goat anti-rabbit IgG (Beyotime, A0208) and HRP-labelled goat anti-mouse IgG (Beyotime, A0216). For immunofluorescence staining, cells or frozen sections were fixed in 4% PFA and blocked with goat serum at room temperature for 1 h. Primary antibodies were added and incubated at 4 °C overnight. The following antibodies were used: Pax7 (DSHB, 1:100), myosin heavy chain (MHC; DSHB, 1:400), embryonic MHC (eMHC; DSHB, 1:500), laminin (Sigma, 1:500) and TCF12 (Abclonal, 1:300). Secondary antibodies were purchased from Invitrogen and were used according to the manufacturer’s instructions. For Pax7 staining, antigen retrieval was performed using 10 mM trisodium citrate dihydrate, which was incubated for 30 min in a 70 °C water bath. All staining images were captured using a fluorescence microscope.

### Co-immunoprecipitation

Co-immunoprecipitation (Co-IP) experiments were performed as described previously with slight modifications^53^. 5uL of primary antibodies were incubated with Protein-G beads (Invitrogen, USA, 10004D) overnight at 4 °C. C2C12 myoblasts and myotubes were collected and lysed in lysis buffer (Thermo Scientific, 87787) in the presence of protease inhibitors (cOmplete mini, Roche). Cell lysates incubated with Protein-G beads for 2 h at 4 °C for pre-clearing and 1/10 for input, then immunoprecipitations were performed using pre-clearing cell lysates with Protein-G beads-antibodies complex overnight at 4 °C. Beads were then washed three times with PBST (0.1%TritionX-100) and bound proteins were eluted by boiling at 100 °C in 0.1 ml of SDS-PAGE Sample Loading Buffer (Beyotime, P0015L). Primary antibodies used: mouse IgG (Santa, sc-2025), HEB (A-6) (Santa, sc-365980), Myogenin (DSHB, F5D), MYOD (Santa, sc-32758X).

### RNA extraction and RNA-seq analysis

RNA was extracted using TRIzol (Simgen, 5301100); two replicates were used for each sample. RNA-seq data were generated using Illumina sequencing. Clean reads were obtained by removing reads containing adapter and ploy-N and low-quality reads from raw data using FastQC and Trimmomatic (version 0.39). Hisat2 (version 2.1.0)^54^ was used to align reads against the mm10 reference genomic assembly of mouse. The gene read counts were calculated using featureCounts^55^ (version 2.0.1) and normalized to transcripts per kilobase million (TPM), which were annotated using Mus_musculus.GRCm38.102.chr.gtf. Differential gene expression analysis between two groups was performed using the R package DESeq2^56^ (|log2FC | ≥ 1, *P* value < 0.01). The Gene Ontology (GO) and Kyoto Encyclopedia of Genes and Genomes (KEGG) functional enrichment analyses were implemented using the Metascape database (http://metascape.org/gp/index.html) and David (https://david.ncifcrf.gov/). R packages were used for the graphical representation of principal component analysis (PCA) plots and heatmaps^57,58^. Genes with TPM ≥ 1 in at least one sample were defined as detected genes (Supplementary Fig. 3b).

### ATAC-seq data processing and alignment

The ATAC-seq libraries of myogenic cells isolated from the TCF12 conditional knockout (TCF12cKO) and Ctrl mice were prepared as previously described^59^. Briefly, samples were lysed in lysis buffer (10-mM tris-HCl (pH 7.4), 10-mM NaCl, 3-mM MgCl2 and NP-40) for 20 min on ice to prepare the nuclei. The optimised concentration of NP-40 was 0.15% for myoblasts and 0.2% for myotubes. Immediately after lysis, the nuclei were centrifuged at 500 g for 5 min to remove the supernatant. Nuclei were then incubated with the Tn5 transposon and tagmentation buffer at 37 °C for 30 min (Vazyme Biotech). Subsequently, the stop buffer was added directly into the reaction system to end tagmentation. PCR was performed to amplify the library for 12 cycles. After the PCR reaction, the libraries were purified and subjected to sequencing using HiSeq2000 (Illumina) according to the manufacturer’s instructions. Briefly, data were checked using FastQC and trimmed to remove Illumina Nextera adapter sequences using trim_galore with the ‘--q 25, --nextera and --fastqc’ options. Low-quality reads were removed using Trimmomatic with default parameters. Clean data processing and alignment were performed using the ENCODE pipeline using ATAC-seq, and peaks were filtered based on the ENCODE mm10 blacklist (https://www.encodeproject.org/files/ENCFF547MET/). Bowtie2 (version 2.3.5.1)^60^ was used to align the reads to the mm10 using the ‘--no-mixed, --no-discordant, --no-unal, --time and --omit-sec-seq’ options. Samtools^61^ was used to sort and isolate uniquely mapped reads using the ‘view -F 4 -bS’, ‘sort -@ 4’, ‘view -F 524 -f 2’, ‘fixmate -r’ and ‘view-F 1804 -f 2’ options. Picard MarkDuplicates was subsequently used to remove duplicates using the ‘VALIDATION_STRINGENCY = LENIENT’ option. The final BAM files were generated using Samtools with the ‘view -bS -q 30 -F 1804 -f 0 × 2’ option. This resulted in the final aligned, de-duplicated BAM file that was used for all downstream analyses. Peak-calling was performed using MACS2 (version 2.1.1)^62^ using the ‘--shift -75 --extsize 150 --nomodel -B --SPMR --keep-dup all’ option (Supplementary Data 4). All alignment results were converted to coverage bigwig files and normalized to the corresponding input using deepTools (version 3.0.2)^63^. R packages were used for the graphical representation of correlation analysis of these samples, which showed highly reproducible results (Supplementary Fig. 4a). Therefore, the replicate files were merged for subsequent analysis. Specific peaks of each sample were also illustrated using merged files (Supplementary Fig. 4b). Furthermore, the ‘findMotifsGenome’ function of HOMER (version v3.1)^64^ was used to identify motifs significantly enriched in open chromatin regions in the wildtype (WT) and TCF12 knockout (TCF12KO) cells, and the ‘mergePeaks’ function was used to overlap the peaks to identify the special and shared peaks among the samples.

### ChIP-seq analysis

Both myoblasts and myotubes of C2C12 cells were used to perform TCF12 ChIP-seq as previously described^17^. The cells were cross-linked using 1% formaldehyde at room temperature, followed by quenching using 200-mM glycine. Sonication was set at 34% power and 20-s ON and 300-s OFF cycles for 6 min. Chromatin was incubated at 4 °C overnight with a TCF12 antibody (HEB (A-6) (Santa, 1:500)). Replicate were also performed. We also downloaded MYOD ChIP-seq data used in a previous study^65^. Clean data were obtained similar to the acquisition of ATAC-seq data as described in the previous section. Bowtie2 was used to align the reads to the reference genome of mouse using the ‘--no-mixed, --no-discordant, --no-unal, --time and --omit-sec-seq’ options. Samtools and Picard MarkDuplicates were subsequently used to sort and isolate uniquely mapped reads and remove duplicates. The final BAM files were generated using Samtools with the ‘view -bS -q 30 -F 1804 -f 0×2’ option. This resulted in the final aligned, de-duplicated BAM file that was used for downstream analyses. For both myoblasts and myotubes TCF12 ChIP-seq data, correlationship analysis was used. Biological replicates of data showed highly reproducible results, 87% and 78% in myoblasts and myotubes, respectively (Supplementary Fig. 4d). Merged bam file was used for downstream analyses. The TCF12 peaks were identified using MACS2 (Supplementary Data 5). The peaks were filtered based on the ENCODE mm10 blacklist. The R package ChIPseeker^66^ was used to identify the nearest genes around the peak and annotate genomic regions of the peaks. deepTools was used for assessing the enrichment profiles of TCF12 ChIP-seq data. All alignment results were then converted to coverage bigwig files and normalized to the corresponding input using deepTools. The bigwig formats can be visualized using the Integrative Genomics Viewer (IGV) software. Peak was adjusted using input data. Profile was used for illustrating the signal of detected binding sites between TCF12 data and input files. Distinct TCF12 binding signals were illustrated by the average intensity profile at TCF12 peak sites comparing with the input files (Supplementary Fig. 4e).

### CUT&Tag data processing and alignment

The CUT&Tag libraries of myogenic cells isolated from the TCF12cKO and Ctrl mice were performed as previously described using MYOD (Santa, sc-32758X)^67^. Cells were counted and 100,000 cells were collected for CUT&Tag library construction using Hyperactive In-Situ ChIP Library Prep Kit for Illumina (Vazyme, TD901-01) according to the manufacturer’s instructions. CUT&Tag data analyses were performed according to the ENCODE transcription factor processing chip-seq pipeline2 (https://github.com/ENCODE-DCC/chip-seq-pipeline2) with parameter of pipeline_type: “ tf”. Genome sequence file and genomic annotation file are similar to ATAC-seq data analysis. Peak-calling was performed using MACS2 (Supplementary Data 6). All alignment results were converted to coverage bigwig files and normalized to the corresponding input using deepTools. R packages were used for the correlationship analysis of these samples with highly reproducible results (Supplementary Fig. 6a). Therefore, the replicate files were merged for subsequent analysis. The R package ChIPseeker was also used to annotate genomic regions of peaks. Peak signal of each sample was also illustrated using merged files (Supplementary Fig. 6b). Furthermore, ‘mergePeaks’ function of HOMER was used to overlap the peaks to identify the special and shared peaks among the samples. For more than 60% shared MYOD peaks were detected between WT SCs MYOD CUT&Tag and C2C12 myoblasts MYOD ChIP-seq data, which showed similar distribution of MYOD binding sites between WT SCs and C2C12 myoblasts.

### Statistics and reproducibility

Statistical analysis was performed using the independent samples Student’s *s*-test (two-sided). Data were presented as mean ± standard error of mean (SEM), *P < 0.05, **P < 0.01, ***P < 0.001. Sample sizes for each experiment were indicated in the relevant results section or figure legends. Statistical analysis was performed in R (v3.4.1) and GraphPad Prism v8 software.

### Reporting summary

Further information on research design is available in the Nature Portfolio Reporting Summary linked to this article.

## Supplementary information

Supplementary Information

Description of Additional Supplementary Files

Supplementary Data 1

Supplementary Data 2

Supplementary Data 3

Supplementary Data 4

Supplementary Data 5

Supplementary Data 6

Supplementary Data 7

Reporting Summary

## Data Availability

RNA-seq, ATAC-seq and TCF12 ChIP-seq and CUT&Tag data that support the findings of this study are available in the SRA database under the accession codes PRJNA749910 and PRJNA749908 (Supplementary Data 1). Accession codes for the published data in GEO used in this study are as follows: MYOD ChIP-seq data (from Cao Y et al.)^65^ are available in the GEO database under the accession code GSE20059. And C2C12 H3K27ac ChIP-seq data^48^ are available in the GEO database under the accession code GSE161056. The uncropped images of Western blot experiments are available in Supplementary Fig. 7. The source data of bar graphs are stored in Supplementary Data 7.

## Code availability

All the codes used in of this study were availability. FastQC, Trimmomatic, Hisat2 and featureCounts were used for RNA-seq analysis. And all the ATAC-seq (https://github.com/ENCODE-DCC/atac-seq-pipeline) ChIP-seq and CUT&Tag (https://github.com/ENCODE-DCC/chip-seq-pipeline2) data analyses were performed according to the ENCODE pipeline. R (v.3.6.1) were used to perform all data visualizations and statistical analyses. All software and R packages used are open source and described in the Methods section with detail parameters.
