# Supplementary material for: Tcf12 is required to sustain myogenic genes synergism with MyoD by remodelling the chromatin landscape
Source: Commun Biol. 2022 Nov 9;5:1201. doi: 10.1038/s42003-022-04176-0 (PMC9646716; doi:10.1038/s42003-022-04176-0)
Supplement: Supplementary file 1 — Supplementary Information [file 42003_2022_4176_MOESM1_ESM.pdf]

# Supplementary figure

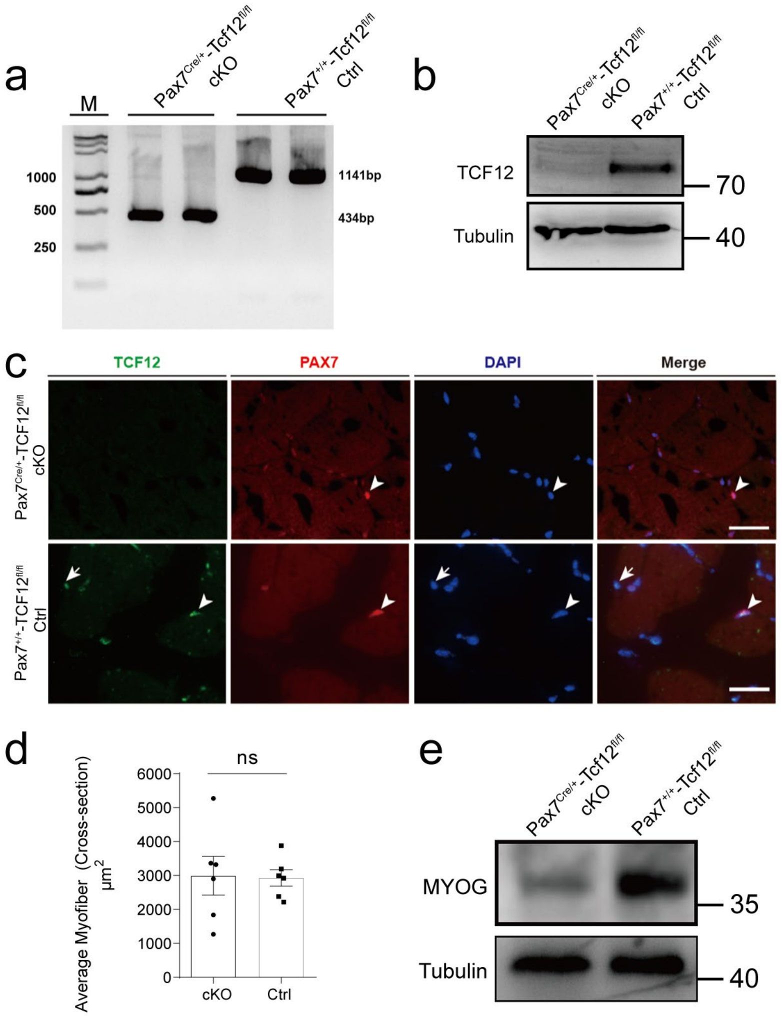


**Supplementary Figure 1. Genotyping and IF staining validation of TCF12cKO mice.** (a) Genotyping PCR in TCF12cKO mice. Mouse DNA was harvested to detect TCF12-floxed and WT alleles, yielding 0.434-kb and 1.1-kb fragments, respectively; (b) Western blot showing relative levels of TCF12 in TCF12cKO and Ctrl mice; (c) IF staining for TCF12 and Pax7 on TA muscles of TCF12cKO and Ctrl mice show that TCF12 protein is absent in both Pax7$^+$ MuSCs and Pax7$^-$ myonuclei (n = 6); arrowheads point to the Pax7$^+$ cells; arrows point to the Pax7$^-$ cells; (d) Quantification of average cross-section area (CSA) of myofiber is calculated, which showed similar average size of myofiber between TCF12cKO and Ctrl mice in TA muscle (n = 6); (e) Western blot showing relative levels of MYOG of limb muscles in TCF12cKO and Ctrl mice. Scale bars = 100 μm. Data are expressed as the mean ± SEM. n.s., not significant, $^*P < 0.05$, $^{**}P < 0.01$, $^{***}P < 0.001$.

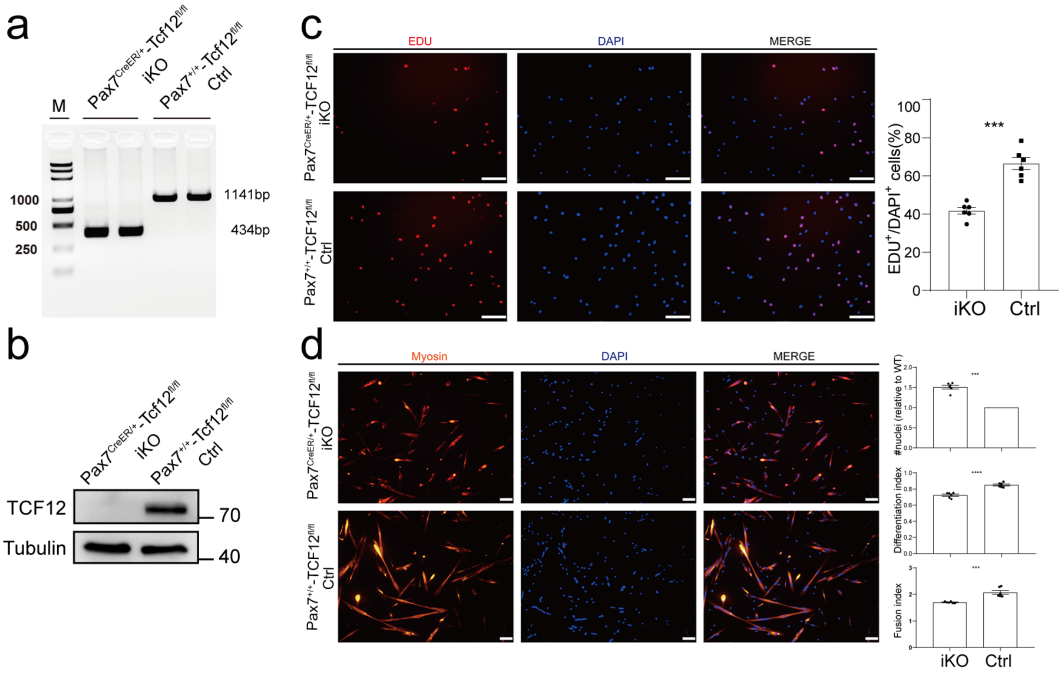


**Supplementary Figure 2. Genotyping and IF staining validation of TCF12iKO mice.** (a) Genotyping PCR in TCF12iKO. Mouse MuSC DNA was isolated after TMX administration to detect TCF12-floxed and WT alleles, which was consistent with the results in TCF12cKO detection; (b) Western blot showing relative levels of TCF12 protein in TCF12iKO and Ctrl mice isolated MuSCs, showing TCF12 protein was diminished in $Pax7^{+}$ progenitors in TCF12iKO mice upon TMX administration; (c) An equal number of isolated MuSCs from TCF12iKO and Ctrl mice were cultured for 24 h and EdU-labeled for 2 h, followed by immunostaining for EdU (red). Quantifications of the percentage of $EdU^{+}$ cells were shown on the right (n = 6); (d) Isolated MuSCs were cultured for 2 days in proliferation medium followed by 2-day differentiation; The extent of differentiation was visualized by IF staining of MYOSIN, and quantified by differentiation and fusion indexes (n = 6). Scale bars = 100 μm. Data are expressed as the mean ± SEM. n.s., not significant, *$P < 0.05$, **$P < 0.01$, ***$P < 0.001$.

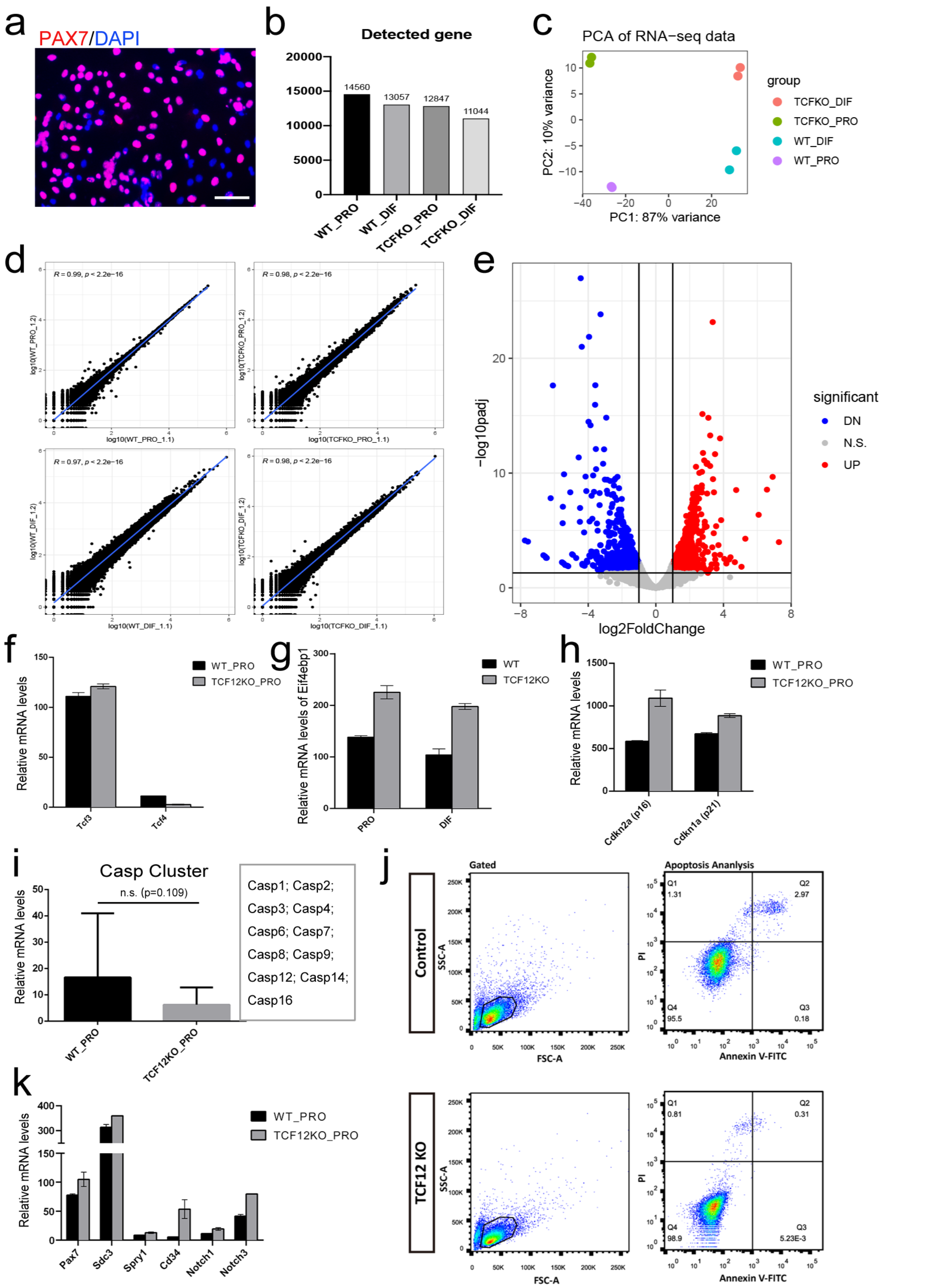

**Supplementary Figure 3. The expression of representative genes in both primary myoblast and myotubes from TCF12 KO and WT controls.** (a) Immunofluorescence (IF) staining for Pax7 and DAPI in the isolated MuSCs of the WT mice. Scale bar = 100 μm; (b) Total number of detected genes (TPM ⩾ 1) in different cells; gene number was similar in

different cells; (c) Principal component analysis of the transcriptome of four cells (WT_PRO, TCFKO_PRO, WT_DIF, and TCFKO_DIF); (d) Correlation analysis of the replicates of four groups of cells, which showed more than 97% correlation for each replicate; (e) Volcano plot of the RNA-seq data of TCFKO_DIF versus WT_DIF cells. Significantly upregulated (red) and downregulated (blue) genes are highlighted (|logFC| > 2, P value < 0.01); (f) Relative mRNA levels of Tcf3 and Tcf4 were examined, which showed that Tcf3 slightly upregulated after Tcf12 ablation; (g) Relative mRNA level of Eif4bp1 was detected using barplot, which showed that Eif4bp1 upregulated after Tcf12 ablation in both myoblasts and myotubes; (h) Relative mRNA levels of senescence marker genes were illustrated between WT_PRO and TCFKO_PRO cells using barplot; (i) Relative mRNA levels of apoptosis marker genes, the Casp gene cluster, were detected using barplot; (j) Apoptosis detection was showed using Flow cytometry. Data analysis was carried out with FlowJo software; (k) Relative mRNA levels of quiescent marker genes, including Pax7, Sdc3, Spry1, and Cd34, even Notch1 and Notch3, were detected using barplot. All the genes were upregulated after TCF12 ablation.

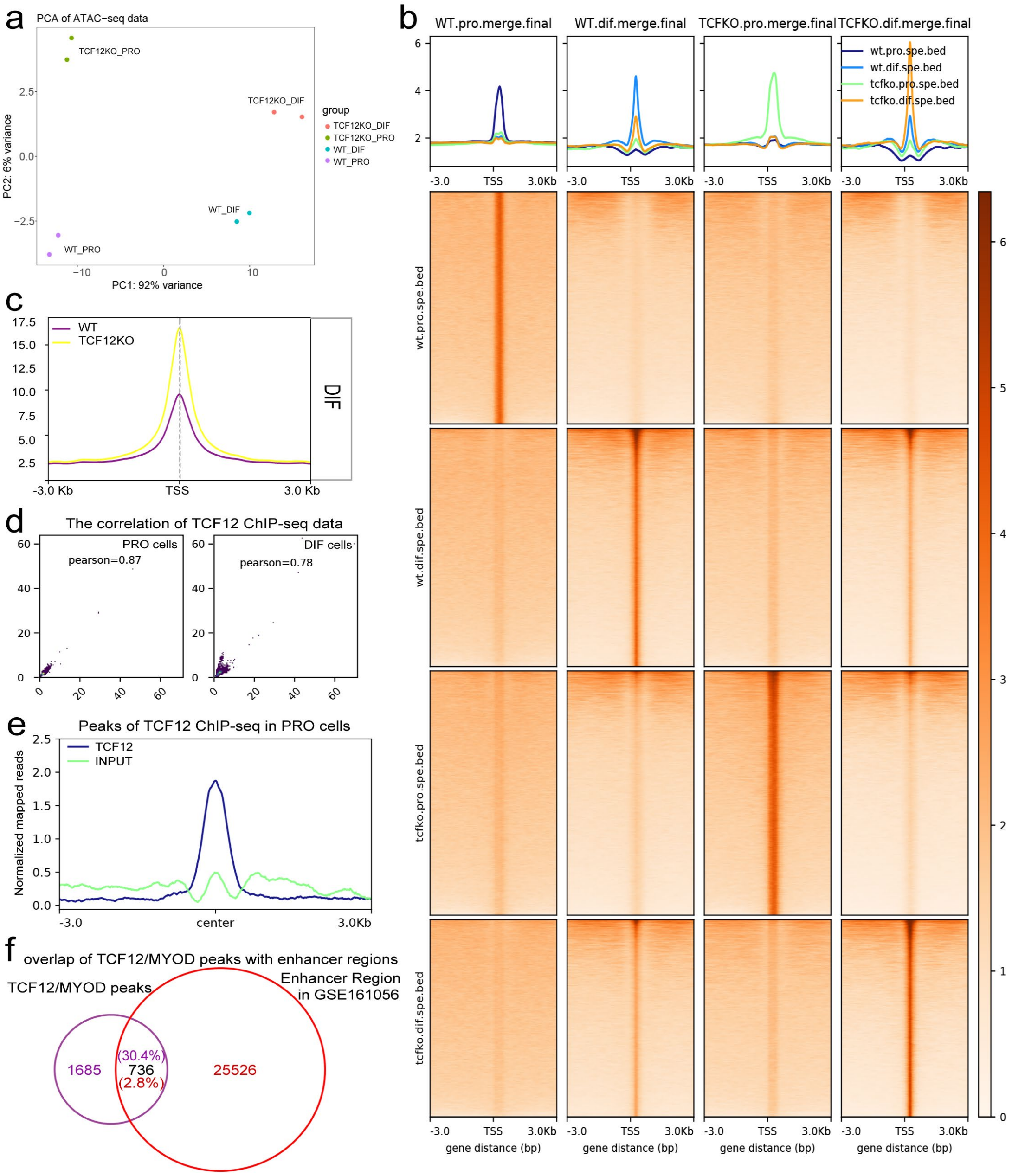


**Supplementary Figure 4. The graphical representation of correlationship and peak signal of the ATAC-seq data and the ChIP-seq data from GEO dataset.** (a) Principal component analysis among the four types of ATAC-seq datasets; (b) Heatmaps and profiles used to illustrate the specific peaks of four cell types; (c) ATAC-seq average signal profiles of 2-day differentiated WT_DIF (purple) and TCFKO_DIF (yellow) cells in the Ctrl and TCF12cKO mice; (d) The correlation of biological replicates of TCF12 ChIP-seq data of proliferation and differentiation cells were showed. Biological replicates of data showed highly reproducible results, 87% and 78% in myoblasts and myotubes, respectively. (e)

Average signal profiles were used to illustrate TCF12 binding signals at TCF12 peak sites comparing with the input files; (f) Venn diagram of the TCF12/MYOD binding sites overlapped with H3K27ac modification of C2C12 downloaded from the GSE161056 assay in GEO dataset. Overlap peaks in TCF12/MYOD peaks (purple) and enhancer region (red) were illustrated; overlapped regions were assigned in grey.

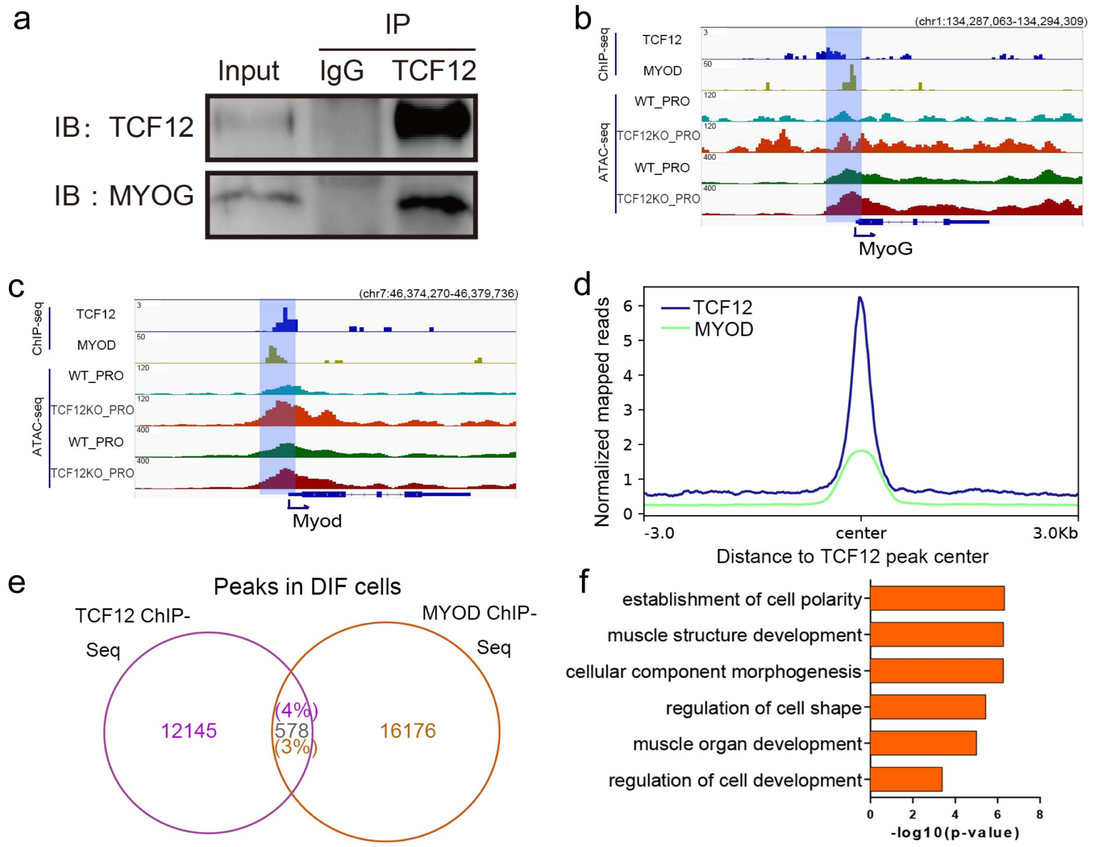


**Supplementary Figure 5. TCF12 does not work together with MYOD in differentiated muscle cells.** (a) Co-IP analysis showing TCF12 interacts with MYOG. Lysates from C2C12 myotubes (60 h) subjected to Co-IP assays with anti-TCF12 or anti-IgG antibodies and blotted with anti-MYOG and anti-TCF12 antibodies; (b and c) IGV browser examples of MyoD and MyoG bound by TCF12 and MYOD. Genome tracks of TCF12 and MYOD ChIP-seq (top) and ATAC-seq of WT_PRO and TCFKO_PRO cells (bottom); (d) Average signal profiles of TCF12 (ratio to input; blue) and MYOD (ratio to input; green) ChIP-seq intensities at TCF12 peaks in terminal differentiated myotubes; (e) Venn diagram representing the overlap of TCF12 and MYOD ChIP-seq peaks of terminal differentiated myotubes. Overlapped peaks in TCF12 peaks (purple) and MYOD peaks (orange) were illustrated; overlapped regions were assigned in grey; (f) GO analysis of the genes associated with these lost MYOD binding sites in TCF12KO cells.

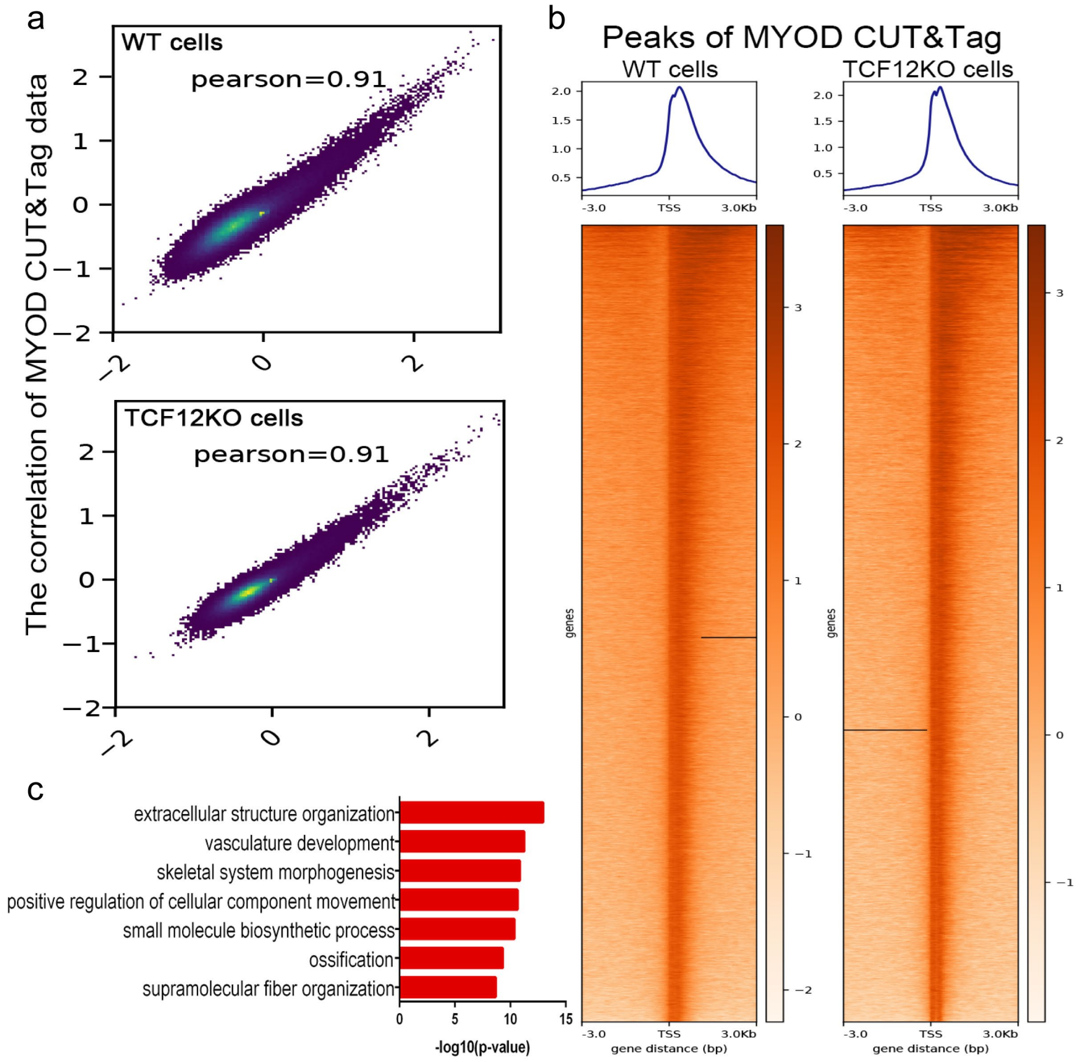


**Supplementary Figure 6. The graphical representation of correlationship and peak signal of the CUT&Tag data.** (a) The correlation of biological replicates of MYOD CUT&Tag data in WT (top) and TCF12KO (bottom) myoblasts. Biological replicates of data showed highly reproducible results, upper than 90%. (b) Heatmaps and profiles used to illustrate the detected MYOD CUT&Tag peaks (ratio to input) in WT (left) and TCF12KO (right) myoblasts; (c) GO analysis of the upregulated genes marked with elevated chromatin accessibility upon TCF12 ablation.

-

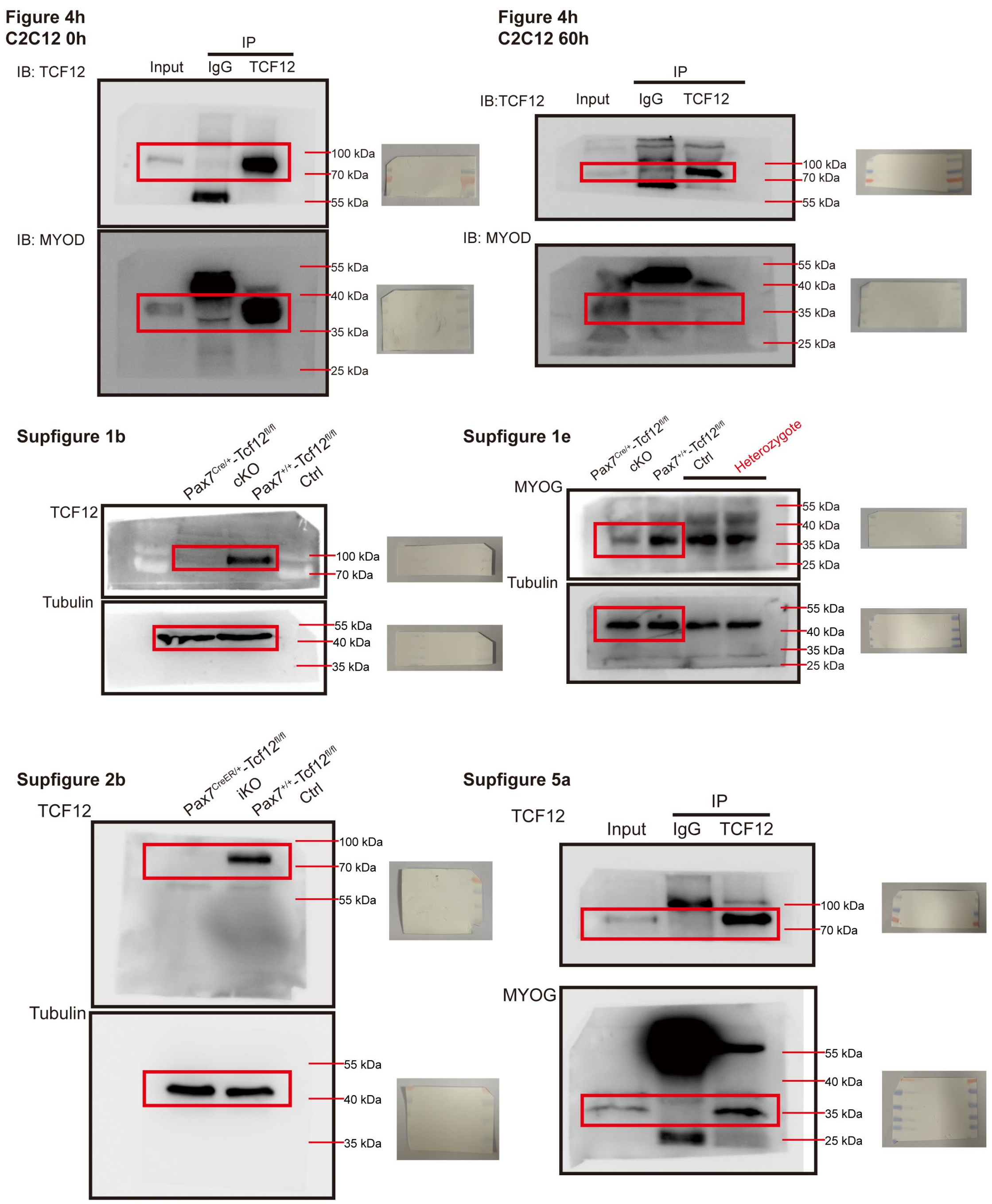


**Supplementary Figure 7. Uncropped original blots in main figures. Heterozygote: heterozygote of littermates for control.**
