# Supplementary material for: Tcf12 is required to sustain myogenic genes synergism with MyoD by remodelling the chromatin landscape
Source: Commun Biol. 2022 Nov 9;5:1201. doi: 10.1038/s42003-022-04176-0 (PMC9646716; doi:10.1038/s42003-022-04176-0)
Supplement: Supplementary file 2 — Description of Additional Supplementary Files [file 42003_2022_4176_MOESM2_ESM.pdf]

Description of Additional Supplementary Files

File name: Supplementary Data 1

Description: Summary of sequencing data from ChIP-seq, ATAC-seq and RNAs-seq.

File name: Supplementary Data 2

Description: Expression level of specific gene list of different cell types

File name: Supplementary Data 3

Description: Expression level of DEGs between TCFKO_PRO and WT_PRO cells

File name: Supplementary Data 4

Description: ATAC-seq peak of different cell types

File name: Supplementary Data 5

Description: TCF12 ChIP-seq peakset

File name: Supplementary Data 6

Description: MYOD CUT&Tag peakset

File name: Supplementary Data 7

Description: Source data of charts in main figures.
