# Supplementary material for: Tcf12 is required to sustain myogenic genes synergism with MyoD by remodelling the chromatin landscape
Source: Commun Biol. 2022 Nov 9;5:1201. doi: 10.1038/s42003-022-04176-0 (PMC9646716; doi:10.1038/s42003-022-04176-0)
Supplement: Supplementary file 10 — Reporting Summary [file 42003_2022_4176_MOESM10_ESM.pdf]

nature portfolio

Corresponding author(s): COMMSBIO-22-3731B

Last updated by author(s): Sep 22, 2022

Reporting Summary

Nature Portfolio wishes to improve the reproducibility of the work that we publish. This form provides structure for consistency and transparency in reporting. For further information on Nature Portfolio policies, see our Editorial Policies and the Editorial Policy Checklist.

Statistics

For all statistical analyses, confirm that the following items are present in the figure legend, table legend, main text, or Methods section.

n/a	Confirmed	
☐	☒	The exact sample size (*n*) for each experimental group/condition, given as a discrete number and unit of measurement
☐	☒	A statement on whether measurements were taken from distinct samples or whether the same sample was measured repeatedly
☐	☒	The statistical test(s) used AND whether they are one- or two-sided<br>*Only common tests should be described solely by name; describe more complex techniques in the Methods section.*
☐	☒	A description of all covariates tested
☐	☒	A description of any assumptions or corrections, such as tests of normality and adjustment for multiple comparisons
☐	☒	A full description of the statistical parameters including central tendency (e.g. means) or other basic estimates (e.g. regression coefficient) AND variation (e.g. standard deviation) or associated estimates of uncertainty (e.g. confidence intervals)
☐	☒	For null hypothesis testing, the test statistic (e.g. *F*, *t*, *r*) with confidence intervals, effect sizes, degrees of freedom and *P* value noted<br>*Give P values as exact values whenever suitable.*
☒	☐	For Bayesian analysis, information on the choice of priors and Markov chain Monte Carlo settings
☐	☒	For hierarchical and complex designs, identification of the appropriate level for tests and full reporting of outcomes
☒	☐	Estimates of effect sizes (e.g. Cohen's *d*, Pearson's *r*), indicating how they were calculated

Our web collection on statistics for biologists contains articles on many of the points above.

Software and code

Policy information about availability of computer code

Data collection	Published ChIPseq was downloaded using stratoolkit version 2.8.0. software
Data analysis	Trimmomatic (version 0.39), Hisat2 (version 2.1.0), featureCounts (version 2.0.1) R version 4.1.0, Metascape database (http://metascape.org/gp/index.html) and David (https://david.ncifcrf.gov/), Bowtie2 (version 2.2.3), Samtools (version 0.1.19), MACS2 (version 2.1.1), deepTools (version 3.0.2), bedtools (version 2.25.0), HOMER (version v3.1).

For manuscripts utilizing custom algorithms or software that are central to the research but not yet described in published literature, software must be made available to editors and reviewers. We strongly encourage code deposition in a community repository (e.g. GitHub). See the Nature Portfolio guidelines for submitting code & software for further information.

Data

Policy information about availability of data

All manuscripts must include a data availability statement. This statement should provide the following information, where applicable:

- Accession codes, unique identifiers, or web links for publicly available datasets
- A description of any restrictions on data availability
- For clinical datasets or third party data, please ensure that the statement adheres to our policy

RNA-seq, ATAC-seq and TCF12 ChIP-seq data that support the findings of this study are available in the SRA database under the accession codes PRJNA749910 and PRJNA749908.

Human research participants

Policy information about studies involving human research participants and Sex and Gender in Research.

Reporting on sex and gender	No human research participants involved in this study
Population characteristics	No human research participants involved in this study
Recruitment	No human research participants involved in this study
Ethics oversight	No human research participants involved in this study

Note that full information on the approval of the study protocol must also be provided in the manuscript.

Field-specific reporting

Please select the one below that is the best fit for your research. If you are not sure, read the appropriate sections before making your selection.

☒ Life sciences ☐ Behavioural & social sciences ☐ Ecological, evolutionary & environmental sciences

For a reference copy of the document with all sections, see nature.com/documents/nr-reporting-summary-flat.pdf

Life sciences study design

All studies must disclose on these points even when the disclosure is negative.

Sample size	Biological replicates or triplicates were usually sufficient to capture variability as estimated by pairwise correlations and PCA. Sample sizes for each experiment were indicated in the relevant results section or figure legends.
Data exclusions	no data was excluded.
Replication	Pairwise correlation and visual inspection of Principal Component Analysis (PCA), all attempts at replication were successful.
Randomization	Randomization of samples was not relevant in this study because it is about molecular processes analyzed in vitro.
Blinding	Blinding of samples was not performed.

Reporting for specific materials, systems and methods

We require information from authors about some types of materials, experimental systems and methods used in many studies. Here, indicate whether each material, system or method listed is relevant to your study. If you are not sure if a list item applies to your research, read the appropriate section before selecting a response.

Materials & experimental systems

n/a	Involved in the study	
☐	☒	Antibodies
☐	☒	Eukaryotic cell lines
☒	☐	Palaeontology and archaeology
☐	☒	Animals and other organisms
☒	☐	Clinical data
☒	☐	Dual use research of concern

Methods

n/a	Involved in the study	
☐	☒	ChIP-seq
☐	☒	Flow cytometry
☒	☐	MRI-based neuroimaging

Antibodies

Antibodies used	Antibody name /Company /Catalog number /Dilution-concentration (see also Materials and method section): HEB (A-6) (Santa, sc-365980, 1:500), TCF12 (Abclonal, 1:300), β-tubulin (ABclonal, 1:1000), HRP-labelled goat anti-rabbit IgG (Beyotime, A0208), HRP-labelled goat anti-mouse IgG (Beyotime, A0216), Pax7 (DSHB, 1:100), myosin heavy chain (MHC; DSHB, 1:400), embryonic MHC (eMHC; DSHB, 1:500), laminin (Sigma, 1:500), Myogenin (DSHB, F5D).
Validation	WB antibodies were validated by Immunoblotting and immunofluorescence staining, even Western blots and Co-

Validation	immunoprecipitation were used to check antibodies.<br>TCF12 ChIP antibodies were validated by Western blots. We did not check species specificity, since it was not relevant for our study.

Eukaryotic cell lines

Policy information about cell lines and Sex and Gender in Research

Cell line source(s)	MuSCs were isolated from the hindlimb muscle tissues. C2C12 Cell line was purchased from and authenticated by ATCC.
Authentication	For MuSCs genotyping PCR was used to validate the presence of all alleles.
Mycoplasma contamination	All cell lines were routinely tested for mycoplasma contamination and they were negative.
Commonly misidentified lines<br>(See ICLAC register)	Commonly misidentified cell lines were not used in this study.

Animals and other research organisms

Policy information about studies involving animals; ARRIVE guidelines recommended for reporting animal research, and Sex and Gender in Research

Laboratory animals	Tcf12tm3Zhu (# 024511), Pax7tm1(cre) (#010530) and Pax7tm1cre/ERT2 (#017763) mice were purchased from Jackson Laboratory.
Wild animals	The study did not involve wild animals
Reporting on sex	Both sexes were used in comparable numbers.
Field-collected samples	The study did not involve field-collected samples.
Ethics oversight	This study was conducted in accordance with the protocols approved by the Hubei Province Committee on Laboratory Animal Care: HZAUMO-2021-0188.

Note that full information on the approval of the study protocol must also be provided in the manuscript.

ChIP-seq

Data deposition

☒ Confirm that both raw and final processed data have been deposited in a public database such as GEO.

☒ Confirm that you have deposited or provided access to graph files (e.g. BED files) for the called peaks.

Data access links<br>*May remain private before publication.*	https://dataview.ncbi.nlm.nih.gov/object/PRJNA749908<br>https://dataview.ncbi.nlm.nih.gov/object/PRJNA749910
Files in database submission	Transcriptome: SRR15255101 WT_PRO-1, SRR15255100 WT_PRO-2, SRR15255097 WT_DIF-1, SRR15255096 WT_DIF-2, SRR15255099 TCF12KO_PRO-1, SRR15255098 TCF12KO_PRO-2, SRR15255095 TCF12KO_DIF-1, SRR15255094 TCF12KO_DIF-2.<br>ATAC-seq: SRR15291820 WT_PRO-1, SRR15291819 WT_PRO-2, SRR15291826 WT_DIF-1, SRR15291825 WT_DIF-2, SRR15291818 TCF12KO_PRO-1, SRR15291817 TCF12KO_PRO-2, SRR15291824 TCF12KO_DIF-1, SRR15291823 TCF12KO_DIF-2,.<br>ChIP-seq: SRR15291822 TCF12_PRO-ChIP, SRR19776868 TCF12_PRO-new-2, SRR19776867 TCF12_DIF-new-1, SRR19776866 TCF12_DIF-new-2, SRR19776865 TCF12_DIF-input-1, SRR15291821 TCF12_PRO-Input.<br>CUT&Tag: SRR19776864 MYOD_WT-new-1, SRR19776863 MYOD_WT-new-2, SRR19776862 MYOD_KO-new-1, SRR19776861 MYOD_KO-new-2, SRR19776860 MYOD_input-new-1, SRR19776859 MYOD_input-new-2.
Genome browser session<br>(e.g. UCSC)	No longer applicable.

Methodology

Replicates	Three replicates were used for RNA-seq dataset. Two replicates were used for every ATAC-seq and ChIP-seq dataset.
Sequencing depth	At least 4M.
Antibodies	TCF12 antibody (HEB (A-6) (Santa, 1:500)).
Peak calling parameters	Peak-calling was performed using MACS2 (version 2.1.1) (ref. 40) using the '--shift -75 --extsize 150 --nomodel -B --SPMR --keep-dup all' option.
Data quality	Data was trimmed for adapter contamination. The mapping rate are all above 96%.

Software | Trimmomatic, Hisat2, featureCounts, R version 4.1.0, Bowtie2, Samtools, MACS2, deepTools, bedtools, HOMER.

Flow Cytometry

Plots

Confirm that:

☒ The axis labels state the marker and fluorochrome used (e.g. CD4-FITC).

☒ The axis scales are clearly visible. Include numbers along axes only for bottom left plot of group (a 'group' is an analysis of identical markers).

☒ All plots are contour plots with outliers or pseudocolor plots.

☒ A numerical value for number of cells or percentage (with statistics) is provided.

Methodology

Sample preparation	Cultured cells were digested by EDTA-free trypsin, cells were centrifuged at 300°C for 5 min. After washed with pre-chilled PBS twice, cells were resuspended with 100 μ 1×Binding Buffer. Add 5μL Annexin V FITC and 10 μL PI Staining Solution, cells are mixed gently and then reacted at room temperature for 15 min avoiding light. Add 400 μL of 1×Binding Buffer, cells are mixed and placed on ice.
Instrument	BD FACS Aria II SORP
Software	FlowJo software was used
Cell population abundance	N/A
Gating strategy	Gating strategy was shown in Supplementary Figure 3J.

☒ Tick this box to confirm that a figure exemplifying the gating strategy is provided in the Supplementary Information.
